# Whole-organ analysis of TGF-β-mediated remodelling of the tumour microenvironment by tissue clearing

**DOI:** 10.1038/s42003-021-01786-y

**Published:** 2021-03-05

**Authors:** Shimpei I. Kubota, Kei Takahashi, Tomoyuki Mano, Katsuhiko Matsumoto, Takahiro Katsumata, Shoi Shi, Kazuki Tainaka, Hiroki R. Ueda, Shogo Ehata, Kohei Miyazono

**Affiliations:** 1 Department of Molecular Pathology, Graduate School of Medicine, The University of Tokyo, Tokyo, Japan; 2 Department of Systems Pharmacology, Graduate School of Medicine, The University of Tokyo, Tokyo, Japan; 3 Laboratory for Synthetic Biology, RIKEN Quantitative Biology Center, Osaka, Japan; 4 Brain Research Institute, Niigata University, Niigata, Japan; 5 Environmental Science Center, The University of Tokyo, Tokyo, Japan

**Keywords:** Cancer imaging, 3-D reconstruction

## Abstract

Tissue clearing is one of the most powerful strategies for a comprehensive analysis of disease progression. Here, we established an integrated pipeline that combines tissue clearing, 3D imaging, and machine learning and applied to a mouse tumour model of experimental lung metastasis using human lung adenocarcinoma A549 cells. This pipeline provided the spatial information of the tumour microenvironment. We further explored the role of transforming growth factor-β (TGF-β) in cancer metastasis. TGF-β-stimulated cancer cells enhanced metastatic colonization of unstimulated-cancer cells in vivo when both cells were mixed. RNA-sequencing analysis showed that expression of the genes related to coagulation and inflammation were up-regulated in TGF-β-stimulated cancer cells. Further, whole-organ analysis revealed accumulation of platelets or macrophages with TGF-β-stimulated cancer cells, suggesting that TGF-β might promote remodelling of the tumour microenvironment, enhancing the colonization of cancer cells. Hence, our integrated pipeline for 3D profiling will help the understanding of the tumour microenvironment.

## Abstract (web summary)

Shimpei Kubota et al. describe a pipeline for quantitative whole-organ analysis that that combines tissue clearing, 3D imaging, and machine learning for analysis of the tumour microenvironment. The authors apply this in a mouse model of lung tumour and reveal the role of TGF-β in remodelling the cellular microenvironment favouring metastatic invasion.

## Introduction

During cancer progression, cancer cells interact with various kinds of non-transformed stromal cells, including fibroblasts, endothelial cells, and immune cells^1^. This group of cellular components is known to constitute the tumour microenvironment. The tumour microenvironment often enhances proliferative and invasive ability of cancer cells. The tumour microenvironment in the metastatic site also determines the establishment of metastasis, which partially accounts for organ tropism of cancer metastasis. Recently, the diversity of the tumour microenvironment was suggested to be involved in cancer progression and resistance to conventional cancer therapy^2,3^. Detailed observation of the tumour microenvironment is thus essential for the development of novel cancer treatment. Although researchers have tried to mimic the interactions between cancer cells and the tumour microenvironments in vivo, methods to monitor the multidimensional structure of cancer are still lacking.

TGF-β is a multifunctional cytokine. TGF-β binds to two types of signalling receptors, and transduces intracellular signals through Smad and non-Smad pathways^4^. TGF-β is important for normal development, as well as progression of many kinds of diseases, including cancers^5^. TGF-β inhibits proliferation of epithelial cells via induction of cyclin-dependent kinase inhibitors and apoptosis-related proteins, or suppression of c-Myc. Mutations or deletions in TGF-β signal components lead to cancer progression, indicating that TGF-β acts as a tumour suppressor^5^. Conversely, in advanced stages of cancer, TGF-β promotes epithelial–mesenchymal transition (EMT) of cancer cells as well as normal epithelial cells, which enhances invasive ability of cancer cells^5^. TGF-β also affects the structure and function of tumour microenvironments; it stimulates the deposition of extracellular matrix, perturbs immune responses, and promotes angiogenesis^6–8^. TGF-β is thus regarded as an important target for the treatment of several types of cancers^9,10^.

Tissue clearing technique has emerged as one of the most powerful strategies for a comprehensive and unbiased analysis of disease progression. Over the past decades, various kinds of tissue clearing methods have been used especially for whole-brain clearing in neuroscience^11,12^. These technologies are widely used in clearing of whole-body or other organs^13–15^ as well as in visualization of cancer cells in vivo^16,17^. We previously established clear, unobstructed brain/body imaging cocktails and computational analysis (CUBIC) and demonstrated its application in cancer research^18,19^. Here, the CUBIC protocol was improved and developed as an integrated pipeline for automated 3D profiling of the tumour microenvironment. We showed its application in the analyses for a mouse tumour model of experimental lung metastasis and explored the role of TGF-β in the tumour microenvironment through profiling of multicellular interactions. Our findings showed several kinds of interactions between cancer cells and the tumour microenvironments that are critical for the metastatic colonization of cancer cells. The application of this enhanced tissue clearing protocol combined with integrated 3D imaging and automated computer analysis will further allow the efficient and accurate probing of the tumour microenvironment.

## Results

### Establishment of one-day tissue clearing protocol for visualization of the tumour microenvironment

**Fig. 1:**
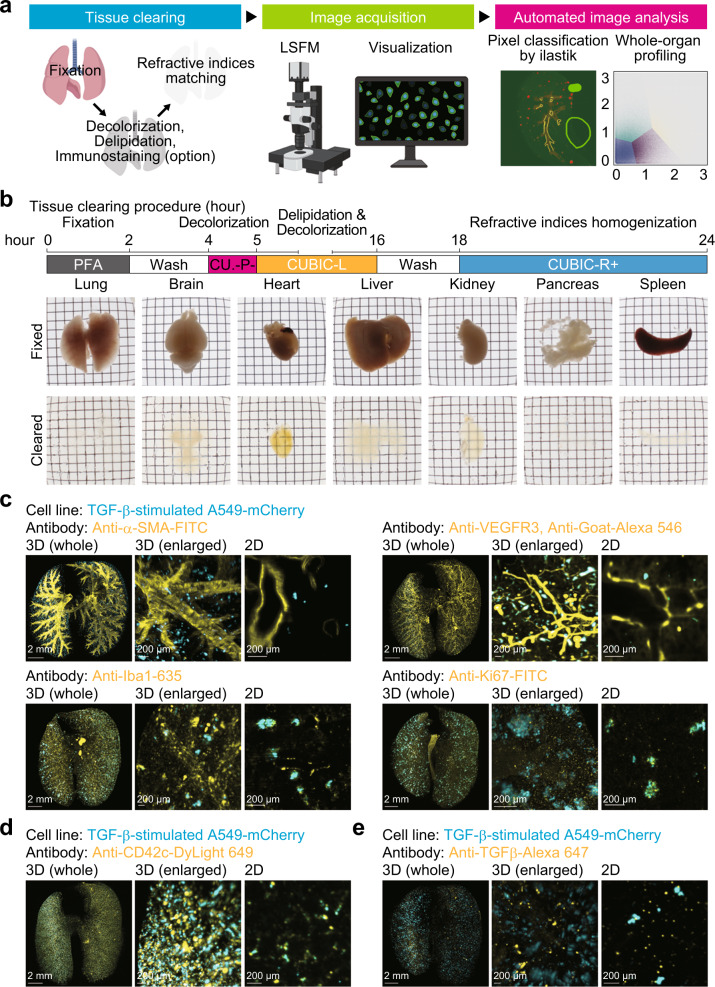
Visualization of the tumour microenvironment using a one-day whole-organ clearing protocol. **a** Scheme of whole-organ profiling of the tumour microenvironment using tissue clearing, image acquisition, and automated image analysis. LSFM light-sheet fluorescence microscope. **b** Protocol of one-day whole-organ clearing (top). Bright-field images of organs (lung, brain, heart, liver, kidney, pancreas, and spleen) after fixation (RI = 1.33) and clearing (RI = 1.52). Fixed organs were stocked in PBS buffer after PFA fixation. **c** Visualization of the tumour microenvironment in the experimental lung metastasis model. A549-mCherry cells were intravenously injected in mice (day 0). Then, the lung was subjected to whole-organ clearing protocol and immunostained with FITC-conjugated anti-α-SMA antibody (day 7), anti-VEGFR3 antibody, Alexa 546-conjugated anti-goat IgG antibody (day 1), Red Fluorochrome (635)-conjugated anti-Iba1 antibody (day 14), or FITC-conjugated anti-Ki67 antibody (day 14). **d** Visualization of the platelets in the experimental lung metastasis model. A549-mCherry cells were intravenously injected in mice (hour 0). Mice were administered with DyLight 649-conjugated anti-CD42c antibody immediately. Then, the lung was subjected to whole-organ clearing protocol, followed by 3D imaging (hour 1). **e** Visualization of TGF-β in the experimental lung metastasis model. A549-mCherry cells were intravenously injected in mice (day 0). Mice were administered with Alexa 647-conjugated anti-TGF-β antibody (day 6). Then, the lung was subjected to whole-organ clearing protocol, followed by 3D imaging (day 7). Representative images are shown. 3D image (whole), scale bar = 2000 μm. 3D image (enlarged), scale bar = 200 μm. 2D image, scale bar = 200 μm. Figure schematic created with biorender.com.

In the present study, we optimized the current tissue clearing protocols (Fig. 1a). Based on comprehensive chemical profiling, three tissue clearing cocktails, CUBIC-P−, CUBIC-L, and CUBIC-R+, were chosen for a one-day tissue clearing protocol. The combination of these clearing cocktails allowed whole-lung clearing in one day (Fig. 1b). Although brain and liver were not delipidated to achieve sufficient transparency for whole-organ profiling, pancreas and spleen became almost completely clear in one day (Fig. 1b), suggesting that this clearing protocol may be available for rapid 3D analysis of cancer metastasis in these organs.

Next, we applied this clearing protocol to the examination of lung metastasis. In this model, mCherry-expressing human lung adenocarcinoma cells A549 were intravenously injected into nude mice. Lungs were extracted from mice and cleared based on this protocol. Microscopic examinations revealed the formation of metastatic colonization of mCherry-positive cells within the entire lungs, as expected (Fig. 1c). At the same time, lungs were immunostained or labelled in vivo using antibodies against protein markers of each cellular component in the tumour microenvironments (Fig. 1c, d): α-smooth muscle actin (α-SMA) for vascular smooth muscle cells, vascular endothelial growth factor receptor-3 (VEGFR3) for lymphatic endothelial cells, ionized calcium binding adapter molecule 1 (Iba1) for activated macrophages, and cluster of differentiation 42c (CD42c) for platelets. As a result, we confirmed that these cellular components of the tumour microenvironment could be visualized with a single-cell resolution in a 3D manner. A marker for cellular proliferation, Ki-67, was also stained in these samples (Fig. 1c). By antibody labelling, we observed TGF-β, one of the cytokines involved in the interactions between cancer cells and the tumour microenvironment (Fig. 1e), throughout the lungs. These results motivated us to use our clearing protocol to visualize potential spatial relationships between cancer cells and the tumour microenvironment.

### Analysis of intercellular distance using whole-organ clearing protocol

**Fig. 2:**
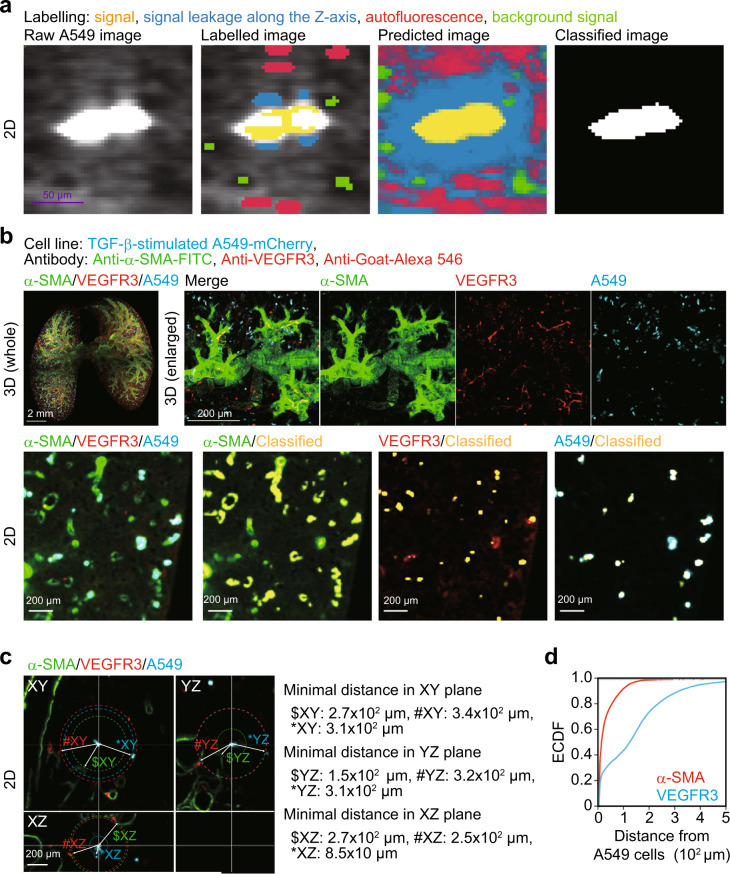
Analysis of intercellular distance using whole-organ clearing protocol. Whole-lung imaging of cancer cells and the tumour microenvironment. A549-mCherry cells were intravenously injected into mice (day 0). Then, the lung was subjected to whole-organ clearing protocol (day 7) and immunostained with anti-VEGFR3 antibody, followed by co-immunostaining with Alexa 546-conjugated anti-goat IgG antibody and FITC-conjugated anti-α-SMA antibody. After pixel classification by ilastik, the original 16-bit images were converted into binary images. **a** The visualization of pixel classification processes. We set four annotations: yellow annotations are true signal, blue annotations are signal leakage along the *Z*-axis, red annotations are tissue autofluorescence, and green annotations are background signal. Scale bar = 50 μm (purple). **b** Representative images. Yellow signals show classified signals of α-SMA, VEGFR3, and mCherry. **c** Quantification of minimal distance from cancer cells to α-SMA, VEGFR3, or mCherry-positive cells. $, #, and * indicate α-SMA, VEGFR3, and mCherry signal at the minimal distance from A549 in 2D plane, respectively. **d** The empirical cumulative distribution function (ECDF) of the minimal distance from cancer cells to α-SMA or VEGFR3 signals. 3D image (whole), scale bar = 2000 μm. 3D image (enlarged), scale bar = 200 μm. 2D image, scale bar = 200 μm. Mouse number in each group is *n* = 3. Representative result of two independent experiments.

To explore interactions between cancer cells and tumour microenvironment, we established an automated image analysis method with pixel classification based on machine learning (Fig. 1a and Supplementary Fig. 1a). In this method, which calculates gaussian smoothing, difference of gaussians, and hessian of gaussian eigenvalues, we do not need to set a threshold value of signal intesnsity and thus are allowed for an unbiased analysis compared to the previous signal intensity-dependent computational method. Using this method, raw images were classified with four annotations and each signal was classified as a component of tumour microenvironment in the lung of mice bearing cancer cells (Fig. 2a, b). To analyse their spatial relationships, minimal distance between each cellular component was measured after the classification (Fig. 2c). The empirical cumulative distribution function (ECDF) showed that 33% of cancer cells localized within 10 μm from α-SMA-positive vessels, and that 24% of cancer cells were within 10 µm from VEGFR3-positive vessels (Fig. 2d). The minimal distance distribution between α-SMA-positive vessels and metastatic lesions was unimodal, whereas that between VEGFR3-positive vessels and metastatic lesions was bimodal (Supplementary Fig. 1b). The density plots showed that 91% of the metastases localized within 100 μm from α-SMA-positive vessels (Supplementary Fig. 1c). Although 43% of the metastases localized within 75 µm from VEGFR3-positive vessels, 53% of the metastases were within 75–400 µm of VEGFR3-positive vessels (Supplementary Fig. 1d). These results suggested that our clearing protocol may provide spatial information on cancer cells and tumour microenvironment instantly when used with automated image analysis methods.

### Metastatic colonization of cancer cells is enhanced in the presence of TGF-β-stimulated cancer cells

**Fig. 3:**
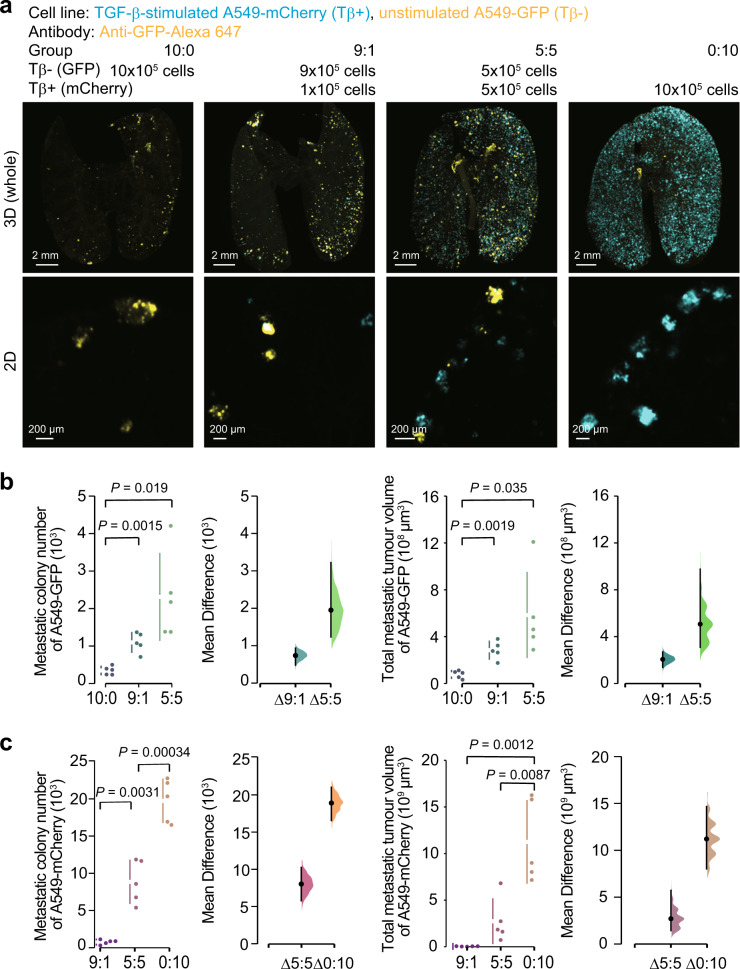
Metastatic ability of a mixed cancer cell population of TGF-β-stimulated and unstimulated cells. Analysis of the cooperative metastasis between the TGF-β-stimulated cancer cells and the unstimulated cancer cells. A549-mCherry or A549-GFP cells were pre-stimulated with or without TGF-β1 for 3 days, respectively, and intravenously injected in mice at the indicated number (day 0). Then, the lung was subjected to whole-organ clearing protocol (day 14), followed by 3D imaging. **a** Representative images. **b** Quantification of the metastatic colony number and the total metastatic tumour volume of A549-GFP cells in the lungs of mice. **c** Quantification of the metastatic colony number and the total metastatic tumour volume of A549-mCherry cells in the lungs of mice. 3D image (whole), scale bar = 2000 μm. 2D image, scale bar = 200 μm. Mouse number in each group is *n* = 5. Representative result of two independent experiments. Data represent the effect size as a bootstrap 95% confidence interval.

Using tissue clearing methods, we previously demonstrated that TGF-β could promote the metastatic colonization of cancer cells through the induction of EMT^19^. To examine whether TGF-β-stimulated cancer cells affect metastasis of other cancer cells within the tumour microenvironment in vivo, both TGF-β-stimulated and unstimulated cancer populations were mixed at various ratios and injected into mice. To distinguish the two cancer populations, prestimulated and unstimulated cancer cells were labelled with different fluorescent proteins, mCherry and green fluorescent protein (GFP), respectively, prior to the determination of their distributions using the clearing protocol and 3D imaging (Fig. 3a). Surprisingly, when 9 × 10^5^ unstimulated cancer cells were injected with 1 × 10^5^ TGF-β-stimulated cancer cells, the number of GFP-positive colonies was larger than that with 1 × 10^6^ unstimulated cancer cells alone (Fig. 3b, group “9:1” vs. “10:0”). Likewise, 5 × 10^5^ TGF-β-stimulated cancer cells further enhanced colonization of 5 × 10^5^ unstimulated cancer cells (Fig. 3b, group “5:5”). These trends were confirmed when the total tumour volumes were determined (Fig. 3b), suggesting that colonization of unstimulated cancer cells was promoted in the presence of TGF-β-stimulated cancer cells. Considering that the total number of injected cancer cells was not different in each group, TGF-β-stimulated cancer cells enhanced colonization of unstimulated cancer cells without affecting the embolization of cancer cells. On the other hand, the number of mCherry-positive colonies was almost proportional to the number of TGF-β-stimulated cancer cells. Unstimulated cancer cells have little influence on the metastatic ability of TGF-β-stimulated cancer cells (Fig. 3c), implying that the effect of TGF-β-stimulated cancer cells on colonization of other populations is commensal.

**Fig. 4:**
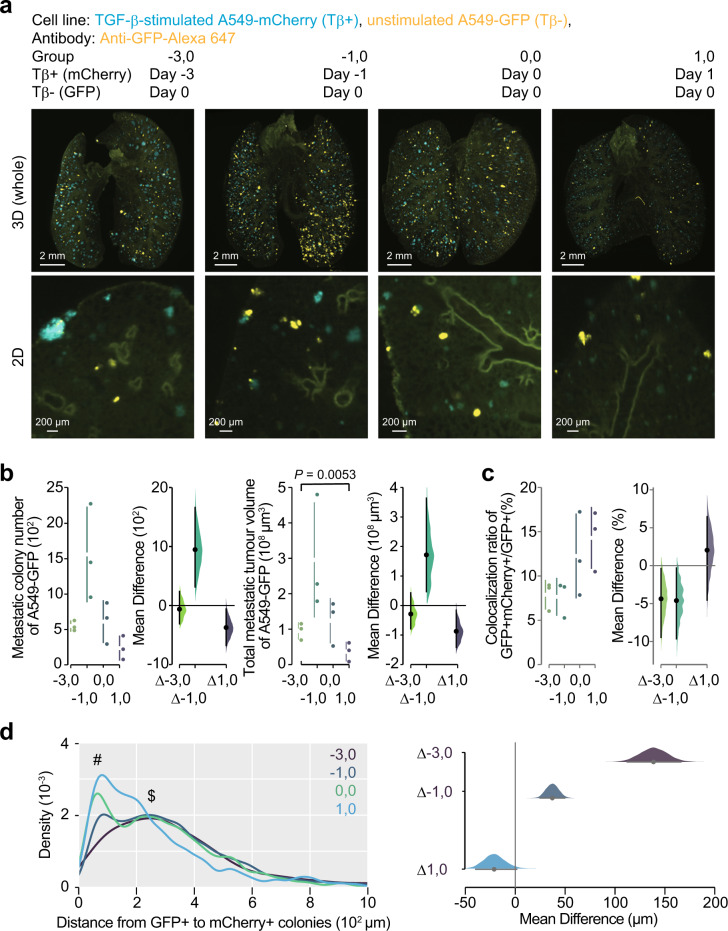
TGF-β-stimulated cancer cells enhance colonization of unstimulated-cancer cells. Temporal analysis of the effect of TGF-β-stimulated cancer cells on the metastasis of unstimulated cancer cells. Unstimulated A549-GFP cells were intravenously injected in mice (day 0). Same number of TGF-β-stimulated A549-mCherry cells were intravenously injected in the same mice at the indicated time point. Then, the lung was subjected to whole-organ clearing protocol (day 14), followed by 3D imaging. **a** Representative images. **b** Quantification of the metastatic colony number and the metastatic tumour volume of A549-GFP cells in lungs of mice. **c** Quantification of the colonies in which unstimulated cells were co-localized with TGF-β-stimulated cells. The ratios of GFP-positive and mCherry-positive colony number to the total GFP-positive colony number were indicated. **d** Density plot of the minimal distance from GFP-positive colony to mCherry-positive colony is shown. # and $ indicate the peaks around 50 µm and 250 µm, respectively. 3D image (whole), scale bar = 2000 μm. 2D image, scale bar = 200 μm. Mouse number in each group is *n* = 3. Representative result of two independent experiments. Data represent the effect size as a bootstrap 95% confidence interval.

Next, we hypothesized that TGF-β-stimulated cancer cells promote colonization of other cancer cells through remodelling of the tumour microenvironment. To investigate this possibility, nude mice were injected with TGF-β-stimulated cancer cells, followed by unstimulated cancer cells after a time lag, or vice-versa (Fig. 4a). When the initial injection of unstimulated cancer cells was followed by an injection of TGF-β-stimulated cancer cells, we could not observe an increase of GFP-positive colony number (Fig. 4b, group “1, 0”). However, when the injection of unstimulated cancer cells was immediately after an initial injection of TGF-β-stimulated cancer cells, GFP-positive colony tended to increase (Fig. 4b, group “0, 0”). Of note, GFP-positive colony number was the highest in mice which were injected with TGF-β-stimulated cancer cells 1 day before the initial injection of unstimulated cells (Fig. 4b, group “−1, 0”). Overall, colonization of TGF-β-stimulated cancer cells preceded the enhancement of colonization of other cancer cells, implying that the effects from TGF-β-stimulated cancer cells might need some time to remodel the tumour microenvironment.

Further, to determine whether TGF-β-stimulated cancer cells enhance colonization of unstimulated cancer cells via direct cancer cell–cancer cell interactions, the distribution of each colony was examined using 3D imaging (Fig. 4c). Only 8% of unstimulated cells contacted TGF-β-stimulated cancer cells in mice in group “−3, 0” and “−1, 0”, whereas 12% of unstimulated cells were adjacent to TGF-β-stimulated cancer cells in mice in group “0, 0”. The distribution of unstimulated cancer cells was further categorized based on the distances from TGF-β-stimulated cancer cells (Fig. 4d). In mice in group “0, 0” and “1, 0”, main population of unstimulated cancer cells was observed in the areas closer to TGF-β-stimulated cancer cells (#). When mice in group “−3, 0” or “−1, 0” were examined, main population of unstimulated cancer cells resided in the areas around 250 μm apart from TGF-β-stimulated cancer cells ($), with only a few cancer cells mutually localized in the vicinity. These results suggest that the promotion of colonization of unstimulated cancer cells by TGF-β-stimulated cancer cells may be due to the remodelling of some components in the tumour microenvironment, rather than direct cancer cell–cancer cell interactions.

### Induction of genes mediating the interactions between TGF-β-stimulated cancer cells and the tumour microenvironment

**Fig. 5:**
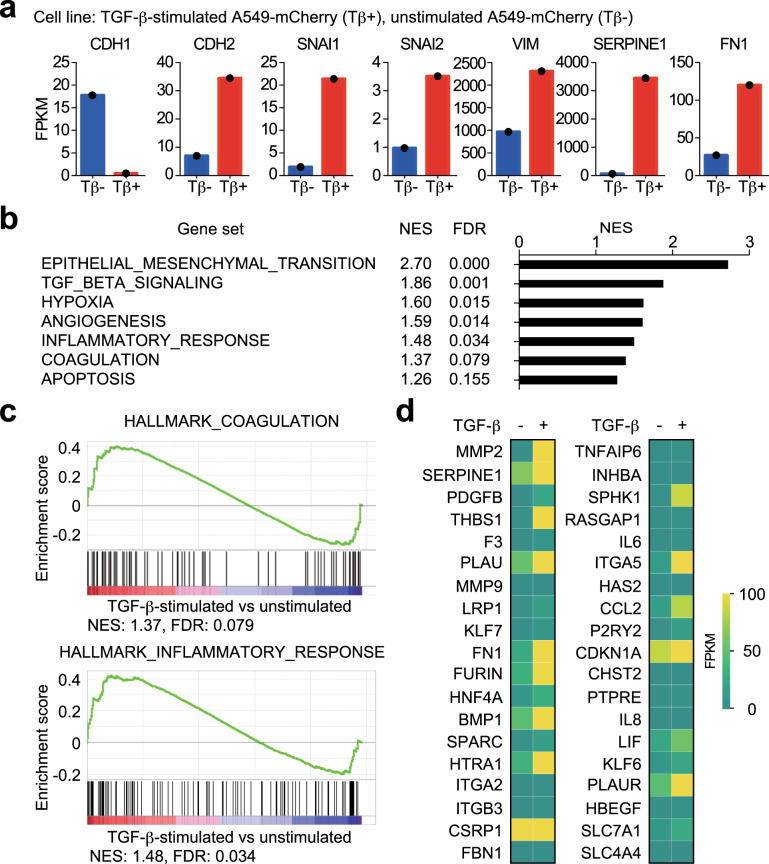
Expression of the tumour microenvironment-related genes in TGF-β-stimulated cancer cells. Identification of targets of TGF-β in A549 cells using RNA-seq analysis. **a** Expression of epithelial-mesenchymal transition (EMT) markers in A549 cells. A549 cells were cultured in the absence (“Tβ−”) or presence of TGF-β1 (“Tβ+”) for 3 days. Then, gene expression level was determined by RNA-seq analysis. Fragments per kilobase of exon per million reads mapped (FPKM) values of each gene are shown. **b**–**d** Gene expression data obtained in **a** were used for gene set enrichment analysis (GSEA). A list of the most enriched hallmark gene in “Tβ+” sets is presented in **b**. The normalized enrichment score (NES) and false discovery rate (FDR) are shown. Representative enrichment plots of the hallmarks are shown in **c**. Heat map showing the expression of genes listed in the hallmarks in **d**. Genes which were extracted in either or both hallmarks are listed.

Next, we tried to identify the molecular mechanisms for the interactions between TGF-β-stimulated cancer cells and the tumour microenvironment. First, targets of TGF-β in A549 cells were determined using RNA-sequencing (RNA-seq) analysis. Expression of an epithelial marker, CDH1, was downregulated by TGF-β. Expression of mesenchymal marker genes, including CDH2, SNAI1, SNAI2, VIM, SERPINE1, and FN1, were upregulated by TGF-β1 (Fig. 5a), suggesting that RNA-seq analysis successfully extracted targets of TGF-β in A549 cells. Gene expression data applied to gene set enrichment analysis (GSEA) demonstrated that several biological processes or cytokine production, other than “EPITHELIAL_MESENCHYMAL_TRANSITION” or “TGF_BETA_SIGNALING”, were altered by TGF-β stimulation (Fig. 5b). For instance, TGF-β activated “COAGULATION” and “INFLAMMATORY_RESPONSE” in A549 cells (Fig. 5c). Of note, many cytokines which activate the tumour microenvironment, including platelet-derived growth factor, B polypeptide (PDGFB), interleukin-6 (IL6), chemokine (C–C motif) ligand 2 (CCL2), and leukaemia inhibitory factor (LIF) were included as targets of TGF-β (Fig. 5d). For IL6 and PDGFB, we observed an increase in expression after 4 h of TGF-β stimulation (Supplementary Fig. 2). Taken together, these data suggested that several cellular components in the tumour microenvironment, e.g., platelets and leukocytes, are activated by cancer cells through induction of both direct-targets and indirect-targets of TGF-β.

### TGF-β-stimulated cancer cells enhance metastatic colonization of cancer cells through platelets

**Fig. 6:**
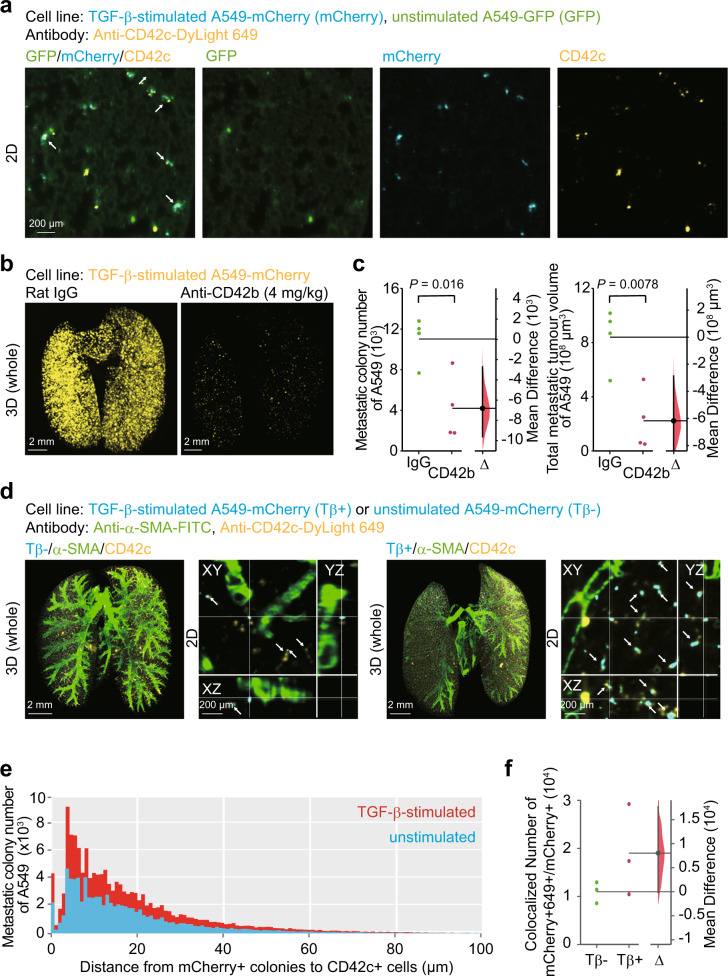
TGF-β-stimulated cancer cells enhance metastasis of unstimulated-cancer cells through platelets. **a** Visualization of cancer cells and platelets in vivo. Mice were pre-treated with DyLight 649-conjugated anti-CD42c antibody. TGF-β-stimulated A549-mCherry and unstimulated A549-GFP cells were injected into nude mice immediately (hour 0). Then, the lung was subjected to whole-organ clearing protocol (hour 1), followed by 2D imaging. The arrows indicate the co-localization of cancer cells and platelets. **b**, **c** Platelet depletion decreased cancer metastasis. Mice were pre-treated with anti-CD42b neutralizing antibody or control IgG. One hour later, TGF-β-stimulated A549-mCherry cells were injected into nude mice (day 0). Then, the lung was subjected to whole-organ clearing protocol (day 14), followed by 3D imaging. Representative images are shown in **b**. Quantification of the metastatic colony number and the metastatic tumour volume of cancer cells are shown in **c**. Mouse number in each group is *n* = 4. Representative result of two independent experiments. **d**–**f** Distribution of the distance between cancer cells and platelets. Mice were pretreated with DyLight 649-conjugated anti-CD42c antibody. TGF-β-stimulated A549-mCherry or unstimulated A549-mCherry cells were injected into nude mice immediately (hour 0). Then, the lung was subjected to whole-organ clearing protocol (hour 1), followed by 3D imaging. **d** Representative images. The arrows indicate co-localization of cancer cells and platelets. **e** A histogram of the minimal distance between cancer cells and platelets. **f** Quantification of the colonies in which stimulated or unstimulated cells were co-localized with platelets. The number of mCherry-positive and DyLight 649-positive colonies is indicated. 3D image (whole), scale bar = 2000 μm. 2D image, scale bar = 200 μm. Mouse number in each group is *n* = 3. Representative result of two independent experiments. Data represent the effect size as a bootstrap 95% confidence interval.

Since RNA-seq analysis suggests a possible relationship between platelets and TGF-β-stimulated cancer cells, contribution of platelets in the mouse tumour model was assessed. Both unstimulated and TGF-β-stimulated cancer cells were injected into mice, which were treated in advance with anti-CD42c antibody for in vivo platelet labelling (Fig. 6a). 2D imaging analysis visualized co-localization of unstimulated or TGF-β-stimulated cancer cells with platelets. To validate the involvement of platelets in metastatic colonization of cancer cells, platelets in mice-bearing cancer cells were depleted by neutralizing antibody against mouse CD42b or rat isotype control antibody (Fig. 6b, c). 3D imaging analysis taken 2 weeks after cancer cell injection demonstrated that both colony number and tumour volume became less than half the original injected number in mice with platelet depletion. Considering that platelet depletion is thought to be sustained for 3 days, platelets might play an important role in the colonization of cancer cells in the early stages of cancer colonization. This time duration was in accordance with the time-course experiments in Fig. 4. Next, the spatial relationship between cancer cells and platelets was examined using 3D imaging (Fig. 6d). Either unstimulated or TGF-β-stimulated cancer cells were intravenously injected into nude mice, which have been treated with antibody for in vivo platelet labelling. Mice lungs were subjected to 3D imaging and immunostaining with anti-α-SMA antibody. Co-localization of cancer cells and platelets was increased in mice bearing TGF-β-stimulated cancer cells, compared to mice bearing unstimulated cancer cells (arrows in Fig. 6d). The distribution patterns of both cells are displayed in Fig. 6e. The number of metastatic colonies distributed within 10 μm from platelets was approximately twice as many in mice bearing TGF-β-stimulated cancer cells as in mice bearing unstimulated cancer cells (Fig. 6f). Overall, these results may suggest that colonization of cancer cells is accelerated by the accumulation of platelets induced by TGF-β-stimulated cancer cells.

### TGF-β-stimulated cancer cells enhance metastatic colonization of cancer cells through the activation of macrophages

**Fig. 7:**
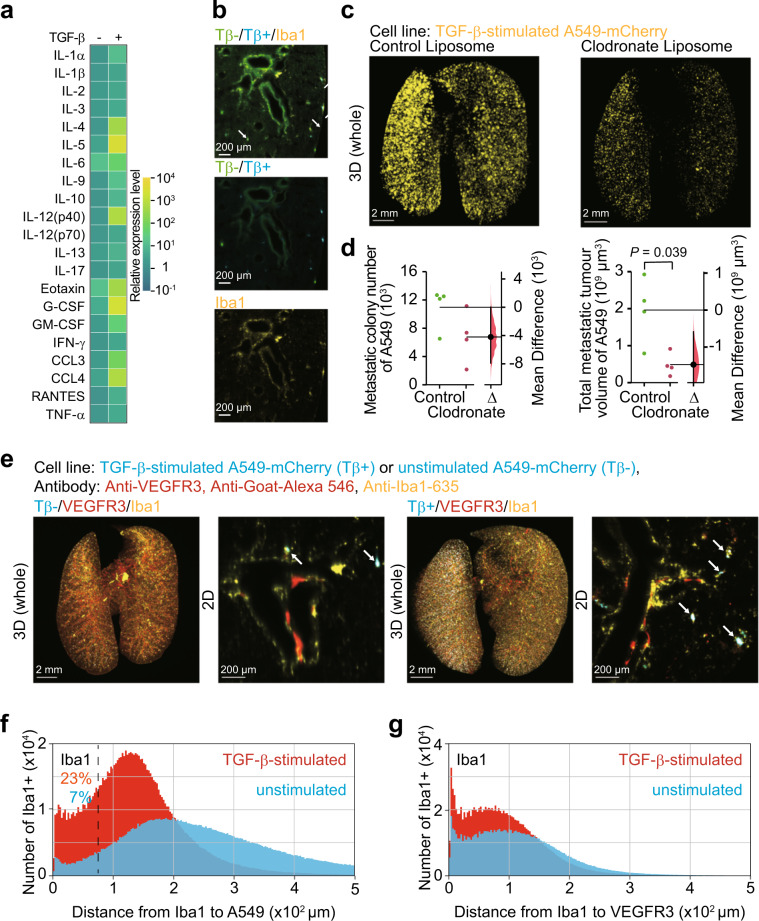
TGF-β-stimulated cancer cells enhance metastasis of unstimulated-cancer cells through the activation of macrophages. **a** Cytokine array of the culture supernatants of the cells obtained from the lung. Unstimulated or TGF-β-stimulated A549-mCherry cells were injected into nude mice. Cells were obtained from the lung of the nude mice 1 day after injection of cancer cells and cultured for one day. **b** Visualization of cancer cells and macrophages. TGF-β-stimulated A549-mCherry (Tβ+) and unstimulated A549-GFP (Tβ−) cells were injected (day 0). Then, the lung was subjected to whole-organ clearing protocol (day 1) and immunostained with anti-Iba1 antibody, followed by 2D imaging. The arrows indicate co-localization of cancer cells and macrophages. **c**, **d** Macrophage depletion decreased cancer metastasis. Mice were pretreated with clodronate or control liposome 3 and 1 day before cancer cell injections. TGF-β-stimulated A549-mCherry cells were injected into nude mice (day 0). Then, the lung was subjected to whole-organ clearing protocol (day 14), followed by 3D imaging. Representative images are shown in **c**. Quantification of the metastatic colony number and the metastatic tumour volume are shown in **d**. Mouse number in each group is *n* = 4. Representative result of two independent experiments. Data represent the effect size as a bootstrap 95% confidence interval. **e**–**g** Distribution of the minimal distance between cancer cells, macrophages, and VEGFR3-positive vessels. TGF-β-stimulated A549-mCherry or unstimulated A549-mCherry cells were injected (day 0). Then, the lung was subjected to whole-organ clearing protocol (day 1), followed by 3D imaging. Representative images are shown in **e**. The arrows indicate the co-localization of cancer cells and macrophages. A histogram of the minimal distance from macrophages to TGF-β-stimulated A549-mCherry cells or unstimulated A549-mCherry cells is shown in **f**. A histogram of the minimal distance from macrophages to VEGFR3-positive vessels is shown in **g**. 3D image (whole), scale bar = 2000 μm. 2D image, scale bar = 200 μm. Mouse number in each group is *n* = 4. Representative result of two independent experiments.

We further confirmed the contribution of TGF-β-induced inflammation to the colonization of cancer cells based on the RNA-seq analysis. We specifically probed for leukocytes, which are activated during TGF-β-induced inflammation through the screening of inflammatory cytokine or chemokine production. Cells were obtained from the lung of mice bearing either unstimulated or TGF-β-stimulated cancer cells. Cytokine array using cell culture supernatants demonstrated that several kinds of inflammatory cytokines and chemokines were produced by cells in the lungs in the presence of TGF-β-stimulated cancer cells (Fig. 7a). Among them, we found that cells from mice bearing TGF-β-stimulated cancer cells specifically produced granulocyte macrophage colony-stimulating factor (GM-CSF) and chemokine (C–C motif) ligand 4 (CCL4), receptors for which are expressed in macrophages. We thus focused our investigation on macrophages in the tumour microenvironments. Co-localization of unstimulated or TGF-β-stimulated cancer cells with macrophages was visualized by 2D imaging analysis (Fig. 7b). Colonization of TGF-β-stimulated cancer cells was diminished in vivo by the treatment with clodronate-containing liposome (Fig. 7c, d). The spatial relationship between cancer cells and macrophages was assessed using 3D imaging (Fig. 7e). Lungs in mice bearing either unstimulated or TGF-β-stimulated cancer cells were examined. 3D imaging demonstrated that co-localization of cancer cells and macrophages was obvious in mice bearing TGF-β-stimulated cancer cells (arrows in Fig. 7e). When the threshold for the distance between cancer cells and macrophages was set as 75 μm, 23% of TGF-β-stimulated cancer cells were close to macrophages. However, only 7% of unstimulated cancer cells distributed similarly (Fig. 7f). Since lymphatic vessels act as tracts for macrophage migration^20^, the proximity of both may suggest that accumulation of macrophages was derived from migration, not from an increase in tissue resident macrophages. Quantitative analysis revealed that 46% of macrophages in mice injected with TGF-β-stimulated cancer cells localized within 75 µm of VEGFR3-positive vessels (Fig. 7g). In contrast, 35% of macrophages from mice injected with unstimulated cancer cells were localized within 75 µm of VEGFR3-positive vessels, implying that TGF-β-stimulated cancer cells may enhance migration of macrophages into the tumour microenvironment. Production of inflammatory cytokines and chemokines in the lung microenvironments is enhanced by TGF-β-stimulated cancer cells, which may activate macrophages and promote the metastatic colonization of cancer cells.

## Discussion

To examine the role of the tumour microenvironment in vivo, a comprehensive analysis of whole organ of mice bearing cancer cells is required. Previously, we developed a tissue clearing method known as CUBIC-Cancer analysis, for whole-body profiling of cancer metastasis^19^. This clearing method is the first-line choice for observing fluorescent reporter proteins in whole-body/organ with a single-cell resolution^21–23^. However, several problems remained. First, the tissue clearing protocol of CUBIC-Cancer analysis requires a relatively long time of 10 days to ensure the transparency of all mouse organs. Such excessive and time-consuming tissue clearing may lead to a reduction in the signal of the fluorescent protein. Through more than 1600 chemical profiling, we selected hydrophilic chemicals for tissue delipidation, decolouring, and refractive index (RI) matching^24,25^. In the present study, three tissue clearing cocktails were used for one-day tissue clearing protocols, using CUBIC-P−, CUBIC-L, and CUBIC-R+. As previously described, CUBIC-L has a decolorizing effect sufficient to reduce light absorption, while CUBIC-P− has a stronger decolorizing effect and shortens the time required for transparency^24^. Second, in our previous protocols, image analysis was performed in a signal intensity-dependent computational method using a commercially available image analysis software. However, image analysis based on the conventional software is susceptible to organ-specific autofluorescence, which increases false-positive classification and the difference of signal intensity at the surface or deeper area of organs. To address these issues, we established automated image analysis methods with pixel classification based on machine learning. In recent years, machine learning has been developed to solve these problems^26,27^. Machine learning tools actually began to be used to analyse 3D images obtained by tissue clearing methods^28–30^. In the present study, we analysed 3D image stacks with ilastik, a machine learning-assisted software for image classification and segmentation^26^. After manual annotations, ilastik calculates the probability of each pixel classified into types of annotations. Binarization based on this probability enabled spatio-temporal analysis of multiple components constituting the tumour microenvironment simultaneously. Here, we provided an integrated pipeline for automated profiling of tumour microenvironment within whole-lung by combining a one-day tissue clearing protocol and automated image analysis with machine learning. Based on the established protocol, we visualized various interactions within the tumour microenvironments in vivo. Our results also suggest that TGF-β evokes coagulation and inflammation through platelets and macrophages, which further enhance the metastatic colonization of cancer cells. In this paper, we quantified the spatial relationship of cellular components in the tumour microenvironment, but it was difficult to quantify the activation of the signalling pathways within the cancer cells. In the future, we believe that the establishment of a reporter system for signalling pathways with a high signal-to-noise ratio will enable us to quantitatively assess the status of cancer cells.

TGF-β is a multifunctional cytokine that regulates many aspects of cellular functions, including cell proliferation, differentiation, apoptosis, migration, and immune surveillance. Among these, TGF-β is known to induce EMT of various kinds of cells. During this process, cancer cells exhibit disruption of tight junctions, loss of cell polarity, increased cell motility, and a spindle-shaped morphology, which allow cancer cells to acquire invasive and metastatic ability^5,31,32^. Using mouse tumour models, we have demonstrated that inhibition of TGF-β signalling prevents metastasis of advanced cancers^33,34^. Only recently, involvement of EMT in cancer metastasis was illustrated in vivo^35–38^. Our previous report demonstrated that EMT promotes not only extravasation, but also survival of cancer cells at metastatic sites^19^. Effects of EMT on colonization of neighboring cancer cells have also been reported. Breast cancer cells which underwent EMT produce Hedgehog ligands in a paracrine manner^39^. This paracrine signal activates GLI-Kruppel family member GLI1 (Gli1) signalling in neighboring cancer cells and promotes their metastasis^39^. In the present study, unstimulated cancer cells were mixed with cancer cells with EMT phenotype by prestimulation with TGF-β. The influence of the mutual colonization was then examined in vivo. TGF-β-stimulated cancer cell populations commensally promoted the colonization of unstimulated cancer cell populations. This effect was more potent in mice with prior administration of TGF-β-stimulated cancer cells. After the remodelling of the tumour microenvironments, including cellular components, such as platelets and macrophages, TGF-β-stimulated cancer cells affected the metastatic ability of neighboring cancer cells.

Activation of EMT affects the numbers and functions of various cells within the tumour microenvironment^40^. EMT enhances interaction between cancer cells with immune cells. Snail-transduced melanoma cells with EMT phenotypes accelerated the metastasis of cancer cells through not only enhanced invasion, but also induction of immunosuppression^41^. Mesenchymal-like breast cancer cells activated macrophages and induced a tumour-associated macrophage (TAM)-like phenotype by GM-CSF, which in turn induced EMT of breast cancer cells through the production of CCL18^42^. In the present study, we focused on platelets and macrophages as key components of the tumour microenvironment for the colonization of cancer cells. Our results of the RNA-seq analysis revealed that expression of genes related to coagulation or inflammation was increased in response to TGF-β (Fig. 5). Some of these genes, such as *PDGFB* and *IL6*, were upregulated immediately after TGF-β stimulation (Supplementary Fig. 2). These results suggest that at least some of these genes are direct targets of TGF-β. Loss of function assays clarified that both platelets and macrophages contributed to the metastatic colonization of cancer cells (Figs. 6 and 7). Also, accumulation of the macrophages was obvious in the mouse bearing TGF-β-stimulated cancer cells, compared with the mouse bearing unstimulated cancer cells. In addition to the direct effect of EMT on invasive ability of cancer cells, TGF-β signalling in cancer cells enhanced their communication with tumour microenvironment, which may support colonization of cancer cells at metastatic sites indirectly. Since we have already reported that *PDGFB* is one of the targets of TGF-β in several types of cancer cells, our current results were not unexpected^43^. It is possible that PDGF-B as well as other cytokines induced by TGF-β may enhance metastatic colonization of A549 cells. Our experiments showed that TGF-β-stimulated cancer cells activate platelets and promote the formation of metastatic colonization. On the other hand, platelets themselves serve as a source of TGF-β, and there might be a malignant feedback cycle in which platelet-derived TGF-β reactivates cancer cells^44^. In that case, breaking this vicious cycle might be important to inhibit cancer metastasis.

## Methods

### Establishment of GFP/mCherry-expressing cancer cells and cell culture

Human lung adenocarcinoma A549 cells were obtained from American Type Culture Collection (ATCC, Manassas, VA)^45^ and validated using STR profiling at the BEX Co Ltd. (Tokyo, Japan). A549 cells were maintained in Dulbecco’s modified Eagle’s medium (#11965; Thermo Fisher Scientific, Waltham, MA) supplemented with 10% fetal bovine serum, 100 U/ml penicillin G, and 100 μg/ml streptomycin. To establish A549 cells stably expressing firefly luciferase and GFP under the CMV promoter, we used a lentiviral expression system (kindly provided by Prof. Hiroyuki Miyoshi, deceased, formerly Keio University, Tokyo, Japan)^46^. A549-Luc2-mCherry cells in the previous study were used as A549-mCherry cells^19^. The signal intensity of mCherry or GFP in A549 cells was not altered by the presence or absence of TGF-β stimulation.

### Reagents

TGF-β1 (240-B, R&D Systems, Minneapolis, MN) was reconstituted in 4 mM HCl and 0.1% bovine serum albumin (BSA, A3983, Sigma-Aldrich, St Louis, MO). For the pre-stimulation with TGF-β, cells were treated with TGF-β1 at a concentration of 5 ng/ml for 3 days. Cells that were cultured in the same amounts of BSA were used as unstimulated control cells.

### Animal experiments

All experiments were approved by and carried out according to the Animal Care and Use Committee of the Graduate School of Medicine, The University of Tokyo. For experimental lung metastasis, female BALB/c *nu/nu* mice (nude mice, 5-week-old, Sankyo Labo Service Corporation, Inc. (Tokyo, Japan)) were intravenously injected with A549 cells (1 × 10^6^ per mouse)^19^, unless otherwise mentioned. For platelet depletion, purified rat monoclonal antibody against mouse CD42b (R300, emfret Analytics, Eibelstadt, Germany) or non-immune rat antibody (IgG) (C301, emfret Analytics) was injected into the tail vein of mouse 1 h before cancer cell injections (1 μg/g). For macrophage depletion, 10 μl/g of anionic clodronate liposome or anionic control liposome (F70101C-AC, FormuMax Scientific, Inc., Sunnyvale, CA) was injected into the tail veins of mice 3 and 1 day before cancer cell injections.

### Preparation of clearing cocktails

Clearing cocktails were composed of five chemicals, which were selected by chemical screening^24^. CUBIC-P− for decolorization was prepared as a mixture of 10 wt% 1-methylimidazole (M0508, Tokyo Chemical Industry Co., Ltd., Tokyo, Japan). CUBIC-L for decolorization and delipidation was prepared as a mixture of 10 wt% polyethylene glycol mono-p-isooctylphenyl ether/Triton X-100 (12967-45, nacalai tesque, Kyoto, Japan) and 10 wt% N-butyldiethanolamine (B0725, Tokyo Chemical Industry Co., Ltd.). CUBIC-R+ for RI adjustment was prepared as a mixture of 45 wt% 2,3-dimethyl-1-phenyl-5-pyrazolone/antipyrine (D1876, Tokyo Chemical Industry Co., Ltd.), 30 wt% nicotinamide (N0078, Tokyo Chemical Industry Co., Ltd.), and 0.5 wt% N-butyldiethanolamine.

### One-day CUBIC protocol for whole-organ clearing

For preparation of whole-organ clearing samples, nude mice were sacrificed by an overdose of isoflurane (099-06571, FUJIFILM Wako Pure Chemical Corporation, Tokyo, Japan). Then, 20 ml of phosphate buffered saline (PBS, pH 7.4) and 30 ml of 4% (w/v) paraformaldehyde (PFA, 162-16065, FUJIFILM Wako Pure Chemical Corporation) in PBS were perfused independently via the left ventricle of the heart. The excised organs were post-fixed in 4% (w/v) PFA at room temperature for 2 h. The fixed organs were washed three times with PBS for 1 h to remove PFA just before clearing. For intense tissue decolorization, the fixed organs were immersed in CUBIC-P− with gentle shaking at 37 °C for 1 h. Subsequently, the fixed organs were immersed in 50% (v/v) CUBIC-L (1:1 mixture of water and CUBIC-L) and further immersed in CUBIC-L with gentle shaking at 37 °C for 11 h. CUBIC-P− and CUBIC-L were refreshed when the cocktail became coloured. After decolorization and delipidation, the organs were washed three times with PBS at room temperature for 2 h. The organs were further immersed in 50% (v/v) CUBIC-R+ (1:1 mixture of water and CUBIC-R+) and then in CUBIC-R+ at room temperature with gentle shaking for 6 h.

### 3D immunostaining

Decolorized and delipidated fixed organs were subjected to immunostaining with diluted antibodies using 1:100 to 1:200 dilutions in the staining buffer composed of 0.5% (v/v) Triton X-100, 0.25% casein (37528, Thermo Fisher Scientific), and 0.01% sodium azide (31208-82, nacalai tesque) for 3 days at room temperature with gentle shaking. The stained samples were washed with PBS three times at room temperature with gentle shaking, and then postfixed with 4% PFA for 1 h at room temperature with gentle shaking before RI adjustment. Antibodies used in immunostaining and sampling days after cancer cell injections are listed in Supplementary Table 1.

### In vivo labelling with antibodies

For in vivo mouse platelet labelling, DyLight 649-labelled antibody against the glycoprotein Ib platelet (GPIb) subunit β (CD42c) of the murine platelet/megakaryocyte-specific GPIb-V-IX complex (X649, emfret Analytics) were injected into the tail vein of mice (0.1 μg/g). One hour after injection of the antibody, the mice were sacrificed. For in vivo TGF-β labelling, anti-TGF-β antibody (clone 1D11.16.8, BE0057, BioXCell, Lebanon, NH) was reacted with 20 equivalent of Alexa-647 NHS ester (A37573, Thermo Fisher Scientific) in 100 mM phosphate buffer pH 8.0, 150 mM NaCl for 2 days at 16 °C. The resulting solution was purified by gel filtration spin columns (7326231, Bio-Rad Laboratories, Inc., Hercules, CA) which was equilibrated with 100 mM phosphate buffer pH 7.0, 150 mM NaCl. The antibody was injected into the tail vein of mouse (10 μg/g) 6 days after cancer cell injections. One day after injection of the antibody, the mice were sacrificed.

### Microscopy

Whole organ images were acquired with two custom-built light sheet fluorescence microscopes (MVX10-LS, developed by Olympus, Tokyo, Japan). Images were captured at 0.63× objective lens (numerical aperture = 0.15, working distance = 87 mm) with digital zoom from 1× to 6.3× zoom. Lasers of 488, 532, 590, and 639 nm wavelength were used for image acquisition. To cover whole organs, the stage was moved both in the lateral direction and axial direction. When the stage was moved to the axial direction, the detection objective lens was synchronically moved to the axial direction to avoid defocusing. RI matched sample was immersed in a mixture of silicon oil HIVAC-F4 (RI = 1.555, Shin-Etsu Chemical Co., Ltd., Tokyo, Japan) and mineral oil (RI = 1.467, M8410, Sigma-Aldrich) during image acquisition. 3D rendered images were visualized and captured with Imaris software (version 8.1.2, Bitplane AG, Zurich, Switzerland) and Imaris Viewer (version 9.5.1, Bitplane AG).

### General data processing

All data processing without pixel classification was performed in Python using custom scripts based on publicly available standard packages comprising Scipy^47^, Numpy^48^, Pandas^49^, scikit-image^50^, Matplotlib^51^, Seaborn (https://seaborn.pydata.org), and h5py^52^ (The HDF Group, 1997–2020). Cells co-localized with other cells were defined as cells, which were close to the center of gravity of other cells within 20 μm. We made the Docker container of the environment for our analysis pipeline. The Docker file and the documentation are available at https://gitlab.com/TGFbeta/kubota_tgfb.git.

### Pixel classification

To avoid false-positive and false-negative pixel classification by filter-based methods, an interactive machine learning-based image analysis, ilastik was applied^26^. This pixel classification workflow returned a probability to pixels based on pixel features and annotations. Gaussian Smoothing of Color/Intensity from 0.3 px to 10 px, Laplacian of Gaussian of Edge from 0.7 px to 10 px, Gaussian Gradient Magnitude from 0.7 px to 10 px, Difference of Gaussians from 0.7 px to 10 px, Structure Tensor Eigenvalues from 0.7 px to 10 px, and Hessian of Gaussian Eigenvalues from 0.7 px to 10 px were selected. Next, four annotations for the training of pixel classification were set. The first annotation meant the true signals from fluorescent proteins or fluorescent chemicals. The second annotation meant the signal leakage along the *Z*-axis. The third annotation meant the strong autofluorescence in bronchi and blood vessels. The fourth annotation meant the weak and non-specific background fluorescence in the lung. To train the classifier, the process of annotating the pixels, evaluating the prediction map, and reannotating the pixels to correct the eventual mistakes were repeated. To assess the reliability of our analytical pipeline, we calculated overlapping segmentations between the reference images annotated by human expert and the images annotated by the proposed machine learning-based methods. We then quantified Sørensen–Dice coefficient, Jaccard similarity coefficient, and Szymkiewica–Simpson coefficient, which are the commonly used metrics for the evaluation of segmentation tasks.

### Measurement of intercellular distances in 3D

To calculate the minimal distance between the cellular components of the tumour microenvironment, we analysed the classified images with the scipy modules in 3D. First, we calculated the center of gravity of the cancer metastases or macrophage cell using the scipy.ndimage.measurements.center_of_mass module in 3D. Next, the minimal distance between the center of gravity and the signal derived from cellular components was calculated using the scipy.spatial.KDTree module in 3D.

### RNA-seq analysis

RNA-seq analysis was performed with Ion Proton, Ion PI Template OT2 200 Kit v3, and Ion PI sequencing 200 Kit v3 (Thermo Fisher Scientific)^53–55^. A549 cells were treated with or without TGF-β1 for 3 days. cDNA libraries were prepared using the RNeasy Mini Kit with the On-Column DNase Digestion Set (QIAGEN, Venlo, The Netherlands), Dynabeads mRNA DIRECT Purification Kit (Thermo Fisher Scientific), and the Ion Total RNA-Seq Kit v2 (Thermo Fisher Scientific). Sequence data were analysed with the GSEA software^56^ and GSEApy (https://github.com/zqfang/GSEApy).

### RNA isolation and quantitative reverse transcription-PCR (qRT-PCR) analysis

Total RNA was extracted with Isogen reagent (Nippon Gene, Toyama, Japan). cDNA was synthesized using PrimeScript II 1st strand cDNA Synthesis Kit (Takara, Otsu, Japan) according to the manufacturer’s protocol. Gene expression was analysed with StepOne Plus Real time-PCR System (Life Technologies, Carlsbad, CA) and Fast SYBR Green Master Mix with ROX (Roche Diagnostics, Tokyo, Japan). The expression level of each gene was normalized to that of hypoxanthine-guanine phosphoribosyltransferase 1 (HPRT1). Primer sequences are shown in Supplementary Table 2.

### Cytokine antibody array

Cytokine antibody array was performed with Bio-Plex Pro Mouse Cytokine 23-plex Assay (M60009RDPD, Bio-Rad Laboratories, Inc.) according to the manufacturer’s protocol. Nude mice were injected with Hanks’ balanced salt solution (HBSS), unstimulated A549-mCherry cells, or TGF-β-stimulated A549-mCherry cells. One day after injection, lungs were excised from mice and ground with BioMasher. Separated cells were cultured on dishes for one day. The culture supernatant was used for the cytokine array. Cytokine concentrations in each group were normalized by dividing by those of cells from mice injected with HBSS.

### Statistics and reproducibility

An estimation plot showed all data points as a swarmplot in the left axes and the effect size as a bootstrap 95% confidence interval in the right axes. The filled curve indicates the resampled mean difference distribution. Unpaired *t*-test with Welch’s correction was used to compare the total volume and the number of lung metastasis for statistical significance. These statistical analyses were performed with DABEST for making an estimation plot^57^, and SciPy for unpaired *t*-test with Welch’s correction^47^. Each in vitro and in vivo experiment was repeated twice or more independently, with similar results obtained, unless otherwise described.

### Reporting summary

Further information on research design is available in the Nature Research Reporting Summary linked to this article.

## Supplementary information

Supplementary Information

Peer Review File

Description of Additional Supplementary Files

Supplementary Data 1

Reporting Summary

## Data Availability

The analysed data in this study are available at GitLab code repository (https://gitlab.com/TGFbeta/kubota_tgfb.git). Raw and processed RNA-seq data are available at Gene Expression Omnibus (GEO) (GSE153468). The source data underlying plots in Figures are provided in Supplementary Data 1. The additional data that support the findings of this study are available from the corresponding author upon reasonable request.

## Code availability

Our analysis programs in this study are available at GitLab code repository (https://gitlab.com/TGFbeta/kubota_tgfb.git).
