# Supplementary material for: Whole-organ analysis of TGF-β-mediated remodelling of the tumour microenvironment by tissue clearing
Source: Commun Biol. 2021 Mar 5;4:294. doi: 10.1038/s42003-021-01786-y (PMC7935961; doi:10.1038/s42003-021-01786-y)
Supplement: Supplementary file 3 — Description of Additional Supplementary Files [file 42003_2021_1786_MOESM3_ESM.pdf]

Description of Additional Supplementary Files

File name: Supplementary Data 1

Description: Source data underlying plots shown in Fig. 2, Fig. 3, Fig. 4, Fig. 5, Fig. 6, Fig. 7, Supplementary Fig. 1, and Supplementary Fig. 2.
