# Supplementary material for: Whole-organ analysis of TGF-β-mediated remodelling of the tumour microenvironment by tissue clearing
Source: Commun Biol. 2021 Mar 5;4:294. doi: 10.1038/s42003-021-01786-y (PMC7935961; doi:10.1038/s42003-021-01786-y)
Supplement: Supplementary file 5 — Reporting Summary [file 42003_2021_1786_MOESM5_ESM.pdf]

nature research

Corresponding author(s): Kohei Miyazono and Shogo Ehata

Last updated by author(s): Dec 24, 2020

Reporting Summary

Nature Research wishes to improve the reproducibility of the work that we publish. This form provides structure for consistency and transparency in reporting. For further information on Nature Research policies, see our Editorial Policies and the Editorial Policy Checklist.

Statistics

For all statistical analyses, confirm that the following items are present in the figure legend, table legend, main text, or Methods section.

n/a	Confirmed	
☐	☒	The exact sample size (*n*) for each experimental group/condition, given as a discrete number and unit of measurement
☐	☒	A statement on whether measurements were taken from distinct samples or whether the same sample was measured repeatedly
☐	☒	The statistical test(s) used AND whether they are one- or two-sided<br>*Only common tests should be described solely by name; describe more complex techniques in the Methods section.*
☒	☐	A description of all covariates tested
☐	☒	A description of any assumptions or corrections, such as tests of normality and adjustment for multiple comparisons
☐	☒	A full description of the statistical parameters including central tendency (e.g. means) or other basic estimates (e.g. regression coefficient) AND variation (e.g. standard deviation) or associated estimates of uncertainty (e.g. confidence intervals)
☐	☒	For null hypothesis testing, the test statistic (e.g. F, t, r) with confidence intervals, effect sizes, degrees of freedom and P value noted<br>*Give P values as exact values whenever suitable.*
☒	☐	For Bayesian analysis, information on the choice of priors and Markov chain Monte Carlo settings
☒	☐	For hierarchical and complex designs, identification of the appropriate level for tests and full reporting of outcomes
☐	☒	Estimates of effect sizes (e.g. Cohen's d, Pearson's r), indicating how they were calculated

Our web collection on statistics for biologists contains articles on many of the points above.

Software and code

Policy information about availability of computer code

Data collection	In imaging, images were captured with two custom-build light sheet fluorescence microscopes (Olympus). In RNA-sequencing analysis, sequencing was performed using the Ion Proton System (Thermo Fisher Scientific).
Data analysis	All statistical analyses in the study were performed with scipy package and DABEST package. In imaging, images were visualized and captured by Imaris software (Bitplane). In pixel classification, images were analyzed with ilastik. In RNA-sequencing analysis, data analysis was performed using GSEA and GSEApy package. We made the Docker container of the environment for our analysis pipeline. The Docker file and the documentation is available at https://gitlab.com/TGFbeta/kubota_tgfb.git.

For manuscripts utilizing custom algorithms or software that are central to the research but not yet described in published literature, software must be made available to editors and reviewers. We strongly encourage code deposition in a community repository (e.g. GitHub). See the Nature Research guidelines for submitting code & software for further information.

Data

Policy information about availability of data

All manuscripts must include a data availability statement. This statement should provide the following information, where applicable:

- Accession codes, unique identifiers, or web links for publicly available datasets
- A list of figures that have associated raw data
- A description of any restrictions on data availability

Raw and processed data of RNA-seq are available at GEO, GSE153468.
https://www.ncbi.nlm.nih.gov/geo/query/acc.cgi?acc=GSE153468
(token; elwhemkgzvuhzav)

Field-specific reporting

Please select the one below that is the best fit for your research. If you are not sure, read the appropriate sections before making your selection.

[x] Life sciences [ ] Behavioural & social sciences [ ] Ecological, evolutionary & environmental sciences

For a reference copy of the document with all sections, see nature.com/documents/nr-reporting-summary-flat.pdf

Life sciences study design

All studies must disclose on these points even when the disclosure is negative.

Sample size	Sample size for each experiment is indicated in the figure legend for each experiment. The sample size was chosen based on previous experience for each experiment to yield high power to detect specific effects. No statistical methods were used to predetermine sample size.
Data exclusions	No samples or analyses were excluded.
Replication	All experimental findings were reliably reproducible.
Randomization	In in vivo experiments, animals were randomly assigned.
Blinding	Blinding of animal studies were not possible due to tagging of animals.

Reporting for specific materials, systems and methods

We require information from authors about some types of materials, experimental systems and methods used in many studies. Here, indicate whether each material, system or method listed is relevant to your study. If you are not sure if a list item applies to your research, read the appropriate section before selecting a response.

Materials & experimental systems

n/a	Involved in the study	
[ ]	[x]	Antibodies
[ ]	[x]	Eukaryotic cell lines
[x]	[ ]	Palaeontology and archaeology
[ ]	[x]	Animals and other organisms
[x]	[ ]	Human research participants
[x]	[ ]	Clinical data
[x]	[ ]	Dual use research of concern

Methods

n/a	Involved in the study	
[x]	[ ]	ChIP-seq
[x]	[ ]	Flow cytometry
[x]	[ ]	MRI-based neuroimaging

Antibodies

Antibodies used

FITC-conjugated anti-α-SMA antibody (F3777, Sigma-Aldrich)
FITC-conjugated anti-Ki-67 antibody (11-5698-82, Invitrogen)
Alexa Fluor 647-conjugated anti-GFP antibody (338006, Biolegend)
Red Fluorochrome(635)-conjugated anti-Iba1 antibody (013-26471, FUJIFILM)
Anti-VEGFR3 antibody (AF743, R&D SYSTEMS)
Alexa Fluor 546-conjugated anti-Goat IgG (H+L) cross-adsorbed secondary antibody (A-11056, Thermo Fisher Scientific)
InVivoMAb anti-mouse/human/rat/monkey/hamster/canine/bovine TGF-β (BE0057, BioXCell)

Validation

Antibodies used for immunoblotting:
FITC-conjugated anti-α-SMA antibody (F3777, Sigma-Aldrich)
Validation: https://en.cellsignal.jp/products/primary-antibodies/nf-kb-p65-d14e12-xp-rabbit-mab/8242

FITC-conjugated anti-Ki-67 antibody (11-5698-82, Invitrogen)
Validation: https://www.thermofisher.com/order/genome-database/dataSheetPdf?producttype=antibody&productsubtype=antibody_primary&productId=11-5698-82&version=111

Alexa Fluor 647-conjugated anti-GFP antibody (338006, Biolegend)
Validation: https://www.biolegend.com/en-us/products/alexa-fluor-647-anti-gfp-antibody-5481

Red Fluorochrome(635)-conjugated anti-Iba1 antibody (013-26471, FUJIFILM)
Validation: https://labchem-wako.fujifilm.com/us/category/01213.html

Anti-VEGFR3 antibody (AF743, R&D SYSTEMS)
Validation: https://www.rndsystems.com/products/mouse-vegfr3-flt-4-antibody_af743

Antibodies used for Neutralization:
InVivoMAb anti-mouse/human/rat/monkey/hamster/canine/bovine TGF-β (BE0057, BioXCell)
Validation: https://bxcell.com/product/m-h-tgf-beta/

Eukaryotic cell lines

Policy information about cell lines

Cell line source(s)	Human lung carcinoma, A549 (American Type Culture Collection, VA) were used in the study.
Authentication	A549 cells were purchased from American Type Culture Collection.
Mycoplasma contamination	Routine mycoplasma testing was performed by PCR regularly on these cells.
Commonly misidentified lines (See ICLAC register)	No commonly misidentified cell lines were used in this study.

Animals and other organisms

Policy information about studies involving animals; ARRIVE guidelines recommended for reporting animal research

Laboratory animals	BALB/c-nu/nu female mice were obtained from Sankyo Labo Service Corporation (Tokyo, Japan).
Wild animals	The study did not involve wild animals.
Field-collected samples	The study did not involve field-collected samples.
Ethics oversight	All experiments were performed under the approval of the Animal Ethics Committee of The University of Tokyo.

Note that full information on the approval of the study protocol must also be provided in the manuscript.
