# Supplementary material for: Whole-organ analysis of TGF-β-mediated remodelling of the tumour microenvironment by tissue clearing
Source: Commun Biol. 2021 Mar 5;4:294. doi: 10.1038/s42003-021-01786-y (PMC7935961; doi:10.1038/s42003-021-01786-y)
Supplement: Supplementary file 1 — Supplementary Information [file 42003_2021_1786_MOESM1_ESM.pdf]

Reviewers' comments:

Reviewer #1 (Remarks to the Author):

Dear Editor,

I've read the manuscript "Whole-organ analysis of TGF-β-mediated remodelling of the tumour microenvironment" by Kubota and co-workers. The Authors describe a complete pipeline for quantitative whole-organ analysis, which encompasses clearing, staining, imaging and image analysis. The potential of the pipeline is demonstrated in a mouse model of lung tumour. Strong – although not conclusive – indications concerning the role of TGF-beta in remodelling the cellular microenvironment favouring metastatic invasion are revealed. This is indeed a nice and convincing demonstration of how quantitative 3D whole-organ analysis could contribute significant advancement in biomedical research. For this reason, I am in favour of publication on "Communications Biology".

However, I found that several points must be addressed prior to final acceptance.

Major points:

1. Quantitative image analysis is one of the cornerstones of this work. Thus, it might be characterized way better. The Authors should provide an error estimate (like precision, recall and F1-score – or other scores) and compare these results with previous methods used by the same authors. Also, evaluation of the accuracy of pixel classification in different samples would guarantee that no bias is introduced by the analysis pipeline. (for instance, the presence of stimulated or unstimulated cells could alter the fluorescence / autofluorescence levels).
2. Inter-cellular distances are computed in 2D or in 3D? This is not clear from figure 2. In general, 3D distance is smaller than minimum distance in 2D slices. Please describe better how distance is computed.
3. Still concerning software: since it is a fundamental piece of the article, it should be available on some repository. I strongly suggest the Authors to upload it on github or on similar platforms.

Minor points:

1. The genes up- or down-regulated by TGF-beta are not necessarily direct targets of this cytokine. Maybe their regulation occurs through some biochemical cascade that is initiated by TGF-beta but requires other components. Although this consideration is maybe obvious for many people in the field, I suggest the Authors to better explain the possibility of direct vs. indirect interaction, given the wide audience of the Journal.
2. Figure 3c: in the graphs, the group is reported as 1:9 instead of 9:1
3. Acronym FDR (figure 5 and legend) is not defined anywhere.
4. I suggest defining the acronym TGF-beta already in the abstract
5. Line 282: "unidirectional" is probably a typo for "unimodal"

Reviewer #2 (Remarks to the Author):

Summary
The authors present an exciting and novel finding of the role of TGFbeta on whole mount lung cancer signaling in a novel imaging modality that illuminates the tumor microenvironment. The authors nicely demonstrate in vitro mechanistic studies of the function and significance of the classical TGFbeta mediate EMT response in human lung cancer cells. Further experiments show that TGFbeta stimulated

A549 lung cancer cells were sufficient for enhanced growth in mouse lungs via cooperation with the host microenvironment. Additionally, the authors show the power of the CUBIC as a visualization tool for whole lung imaging of the tumor microenvironment. Overall, the work advances the ability to visualize and understand the microenvironment which has been reduced to 2D or single cell analyses which limits our spatial and functional understanding of the metastatic process.
I have provided some suggestions necessary to improve the manuscript from its present state:
The title does not accurately describe the nature, purpose, and benefit of the study. I suggest the authors develop a new title that explains the novelty of the lung microenvironment explored with the CUBIC system. The abstract reads much too like a background introduction and does not abstractly tell the reader of the experiments and their impact for the reader. The authors should be direct in that their analysis limited to a single cell line and organ the lung. I enjoy this manuscript and the discussion can be the appropriate place to expand on additional organs, cancer types and signaling pathways that could be analyzed in this novel way.
Specific questions and concerns about the data presented:
Figure 1:
1) Did the authors consider showing side by side comparative H&E to appreciate localization at high power?
2) Does the metastasis number change for cells trapped in large vessels that have not extravasated?
Figure 2:
3) The aSMA stain is difficult to see as two images in 2A.

Figure 3:
4) Can the authors quantitate the metastatic volume in the parenchyma vs vessels? Can micromets vs mets with more than 50 cells be distinguished?

Figure 4:
5) Is there a difference between right or left lungs? Do specific lungs have different regional enhancements? Does the tissue clearing work better for larger or smaller lung tissue?
Figure 5:
6) Were there any unexpected TGFbeta responses or signaling pathways? Do specific known mutations in A549 human lung cancer cells cause TGFbeta response to lose growth control or other switching from tumor suppression to promotion?
Figure 6:
7) Could the authors expand on the platelet mechanism for a source of activated TGFbeta and how this strategy compares with other TGFbeta targeting approaches in greater depth?

Dear Reviewers,

We would like to thank the reviewers for constructive comments to our current work. We have taken all these comments and suggestions into account in the revised version of our paper. We listed one-by-one response below. Revised points in the manuscript - were marked by red.

Reviewer #1 (Remarks to the Author):

Major points:

1. Quantitative image analysis is one of the cornerstones of this work. Thus, it might be characterized way better. The Authors should provide an error estimate (like precision, recall and F1-score – or other scores) and compare these results with previous methods used by the same authors.

We appreciate the reviewer for raising an important point. To measure the similarity between results by manual annotation and this paper's analysis pipeline, several coefficient scores (Sørensen-Dice coefficient, Jaccard similarity coefficient, and Szymkiewicz-Simpson coefficient) were calculated in the revised version (Supplementary Fig. 1a (page 6, line 8-10) and Methods "Pixel classification." section (main text, page 23, line 4-9)), suggesting the quality of our analysis.

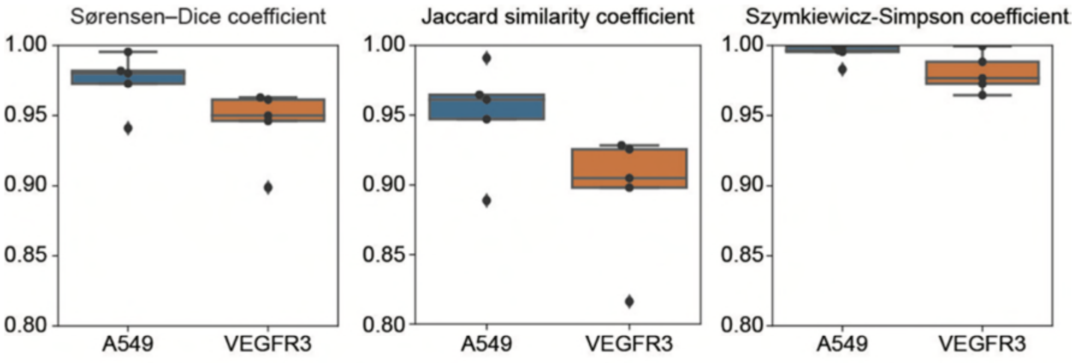

Supplementary Figure 1a

To understand how the proposed machine learning-based methods proceeds, we added these data in the revised manuscript (Fig. 2a (page 6, line 13-16)).

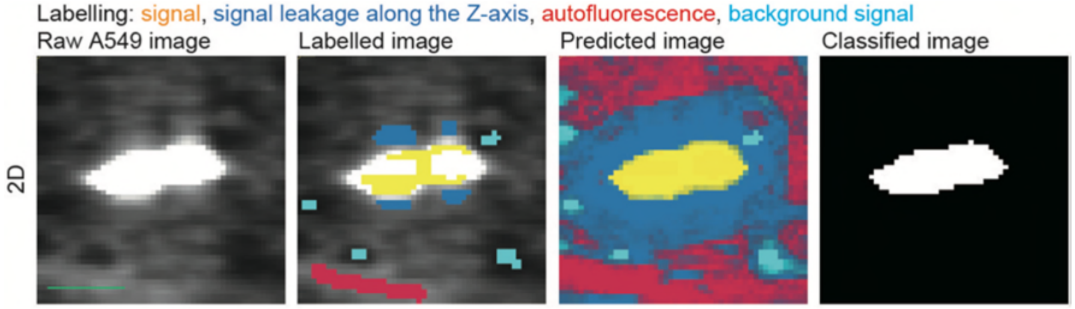

Figure 2a

In the previous paper, we quantify the tumor metastasis with Surface package of Imaris 8.1.2. This previous method could not output the xyz coordinates of the extracted signals and therefore could not perform the quantitative evaluation, as we did in the current method (Supplementary Fig. 1a in the revised version)).

Furthermore, the previous method required a threshold value for signal extraction (Figure for Reviewers 1a). Since the results changed significantly depending on the threshold value setting, we needed to set appropriate thresholds that correspond to differences in signal intensity at the surface area or deep area of tissues. On the other hand, our proposed method, which calculates the Gaussian difference and extracts the signal, doesn’t require to set a threshold value and thus allowed for an unbiased analysis compared to the previous method (Figure for Reviewers 1b). We added these descriptions in the revised manuscript (Results section (main text, page 6, line 10-13) and Discussion section (main text, page 14, line 20-23)).

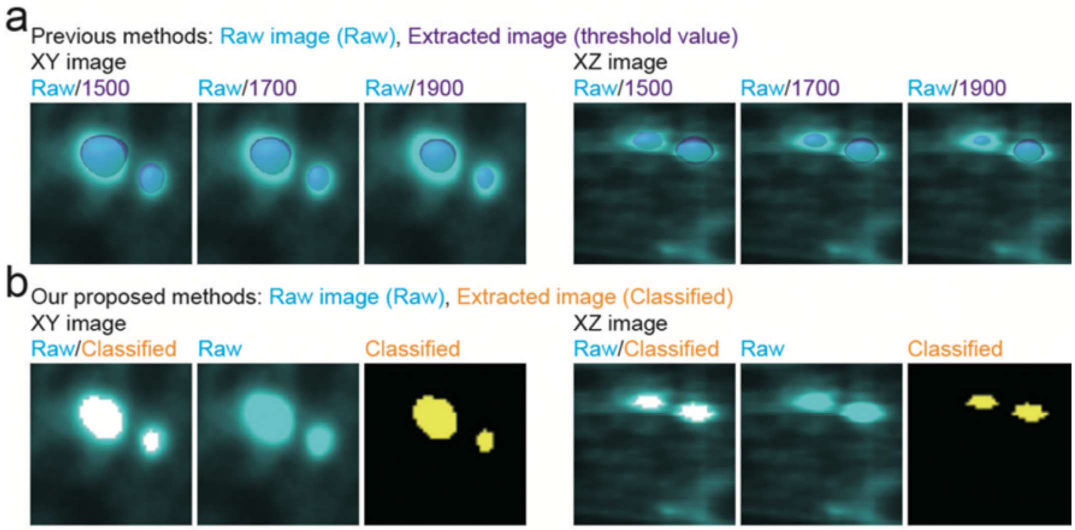

Figure for Reviewers 1

Also, evaluation of the accuracy of pixel classification in different samples would guarantee that no bias is introduced by the analysis pipeline. (for instance, the presence of stimulated or unstimulated cells could alter the fluorescence / autofluorescence levels).

To assess the accuracy of pixel classification in different samples, the fluorescence intensity was compared in TGF-β-stimulated and unstimulated cells. The percentage of cells with GFP signal intensity greater than 10^3 was 99.32% in the TGF-β-stimulated group (up, right) and 96.87% in the unstimulated group (up, middle in Figure for Reviewers 2a). The percentage of cells with mCherry signal intensity greater than 10^3 was 98.53% in the TGF-β stimulated group (down, right) and 98.53% in the TGF-β unstimulated group (down, middle in Figure for Reviewers 2b). These results indicate that signal levels were not altered by the stimulation of TGF-β. We added these descriptions in the revised manuscript (Methods "Establishment of GFP/mCherry-expressing cancer cells and cell culture" section (main text, page 18, line 8-10)).

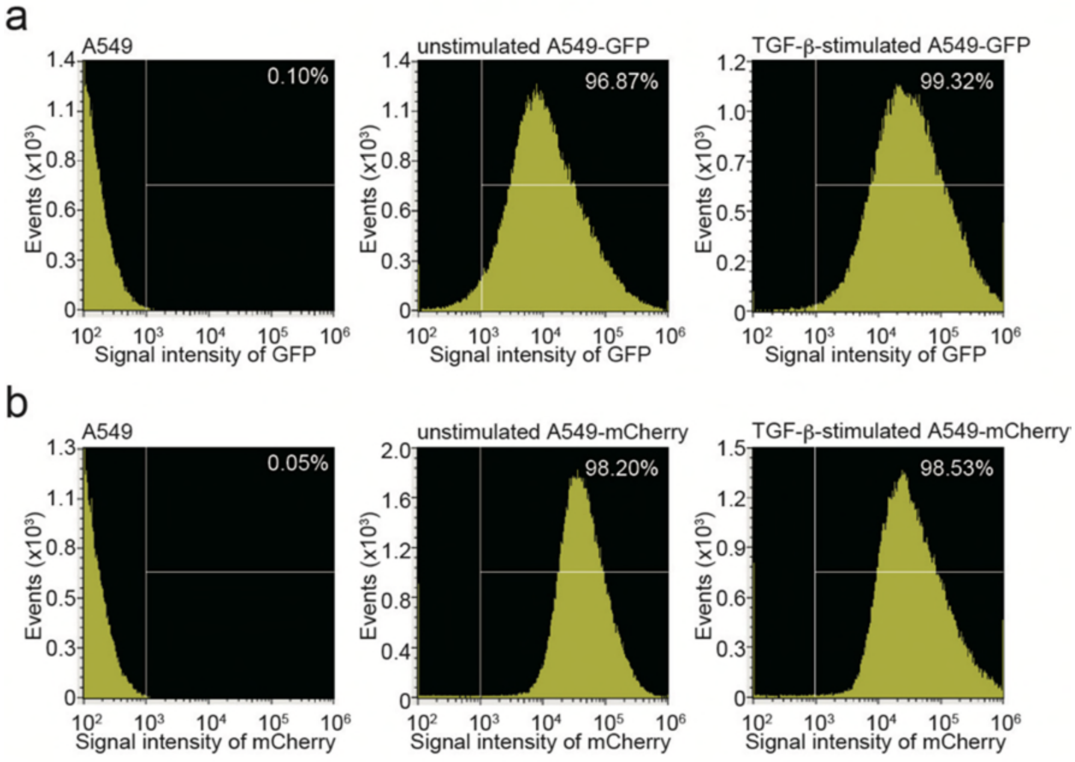

Figure for Reviewers 2

2. Inter-cellular distances are computed in 2D or in 3D? This is not clear from figure 2. In general, 3D distance is smaller than minimum distance in 2D slices. Please describe better how distance is computed.

We appreciate the referee for raising the confusing point. Inter-cellular distances were quantified in 3D. First, the center of gravity of the metastatic colony or macrophage cell was calculated using the scipy.ndimage.measurements.center_of_mass module in 3D. Next, the minimal distance between the center of gravity and the signal for α-SMA or VEGFR3 was measured using the scipy.spatial.KDTree module in 3D. We added these detailed descriptions in the revised manuscript (Methods "Measurement of intercellular distances in 3D" section (main text, page 23, line 11-17)).

3. Still concerning software: since it is a fundamental piece of the article, it should be available on some repository. I strongly suggest the Authors to upload it on github or on similar platforms.

We completely agree with the referee's comments. We made the Docker container of the environment for our analysis pipeline. The Docker file and the Python source code are available at https://gitlab.com/TGFbeta/kubota_tgfb.git. We added these detailed descriptions in the revised manuscript (Materials and Methods "Data and code availability" section (main text, page 25, line 10-15)).

Minor points:

1. The genes up- or down-regulated by TGF-beta are not necessarily direct targets of this cytokine. Maybe their regulation occurs through some biochemical cascade that is initiated by TGF-beta but requires other components. Although this consideration is maybe obvious for many people in the field, I suggest the Authors to better explain the possibility of direct vs. indirect interaction, given the wide audience of the Journal.

According to the referee's suggestion, we investigated whether the up-regulated genes by TGF-β, such as *IL6* and *PDGFB*, are direct targets or indirect targets with time-course analysis. Although some indirect mechanisms further enhance their expression, their up-regulations were initiated 4 hr after the stimulation of TGF-β (Supplementary Fig. 2). These results suggest that at least some of the genes are direct targets of TGF-β. We added these data in the revised manuscript (main text, Results section (main text, page 10, line 8-12) and Discussion section (main text, page 17, line

3-5)).

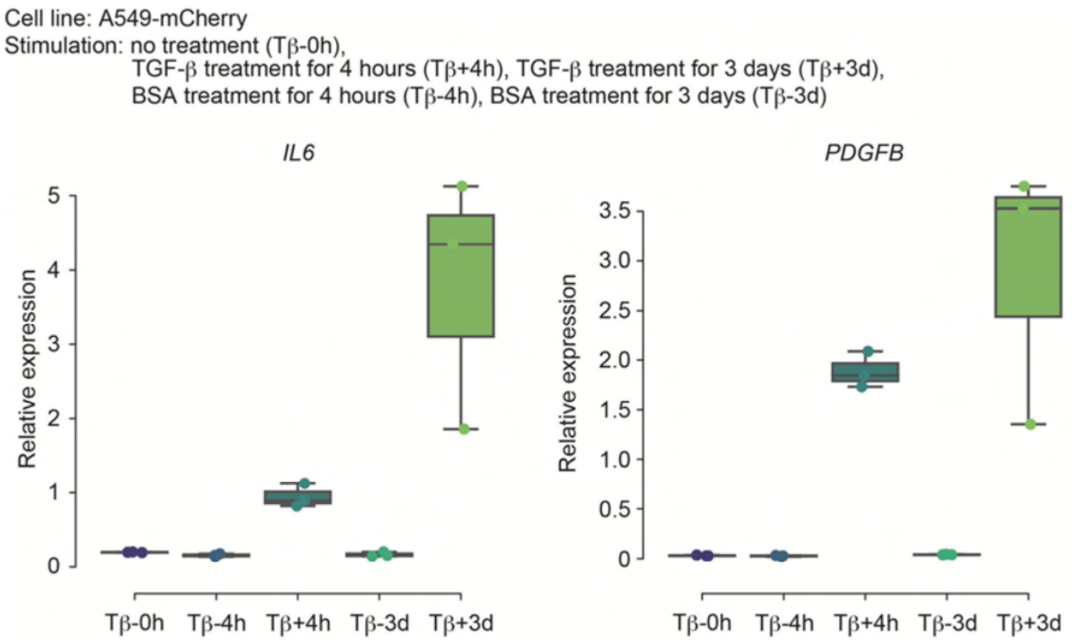

2. Figure 3c: in the graphs, the group is reported as 1:9 instead of 9:1

We appreciate the referee's careful review and comment. We have corrected the description in Fig 3.

3. Acronym FDR (figure 5 and legend) is not defined anywhere.

Acronym for FDR was defined in the legend for Fig. 5, as "The normalized enrichment score (NES) and false discovery rate (FDR) are shown." (main text, page 30, line 9-10).

4. I suggest defining the acronym TGF-beta already in the abstract

Acronym for TGF-β was defined in the abstract, as "We further explored the role of transforming growth factor-β (TGF-β) in cancer metastasis." (main text, page 2, line 6-7).

5. Line 282: "unidirectional" is probably a typo for "unimodal"

Thank you for pointing it out. The typo was corrected in the revised version, as "The

minimal distance distribution between α-SMA-positive vessels and metastatic lesions was unimodal" (main text, page 6, line 20-21).

Reviewer #2 (Remarks to the Author):

Summary

The title does not accurately describe the nature, purpose, and benefit of the study. I suggest the authors develop a new title that explains the novelty of the lung microenvironment explored with the CUBIC system.

We appreciate the referee for raising an important point. To address this concern, we have developed a new title to "Whole-organ analysis of TGF-β-mediated remodelling of the tumour microenvironment by tissue clearing." (main text, page 1, line 1-2)

The abstract reads much too like a background introduction and does not abstractly tell the reader of the experiments and their impact for the reader. The authors should be direct in that their analysis limited to a single cell line and organ the lung.

According to the referee's suggestion, the novelty of our work was emphasized in the abstract in the revised version. We also added the description of our analysis being limited to human lung adenocarcinoma A549 cells and lung metastasis (Abstract section (main text, page 2, line 3-6)).

I enjoy this manuscript and the discussion can be the appropriate place to expand on additional organs, cancer types and signaling pathways that could be analyzed in this novel way.

We appreciate the referee's fruitful suggestion. As we reported in 2017, our analysis pipeline could be adapted to multiple organs, including the brain. We have also successfully used fluorescent proteins, such as GFP and mCherry, as reporters to analyze the metastasis of various cancer cell lines, such as breast cancer (MDA-MB-231 and 4T1), renal cancer (OS-RC-2, Caki-1, and Renca), pancreatic cancer (SUIT-2 and Panc-1), and melanoma (B16F10). In this paper, we quantified the presence or absence of cancer cells, but it was difficult to quantify the extent of signal pathway activation in cancer cells. In the future, we believe that the establishment of a reporter system for signaling pathways with a high signal-to-noise ratio will enable us to quantitatively assess the status of cancer cells. We added these detailed descriptions in the revised

manuscript (Discussion section (main text, page 15, line 13-17)).

Figure 1:

1) Did the authors consider showing side by side comparative H&E to appreciate localization at high power?

We appreciate the referee's comment. H&E staining was performed using serial sections of lung tissues. However, it was difficult to classify cancer cells, macrophages, and platelets even in a high power field (Figure for Reviewers 3). Thus, we did not mention

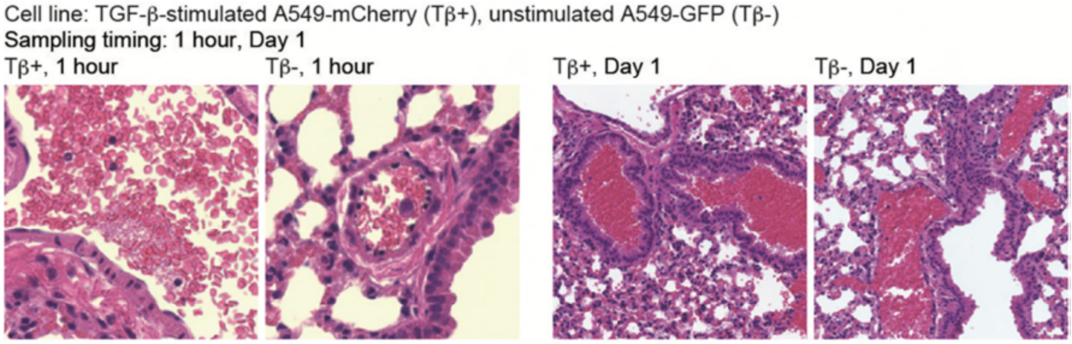

Figure for Reviewers 3

it in the manuscript.

2) Does the metastasis number change for cells trapped in large vessels that have not extravasated?

We appreciate the referee's comment. We have been successful in classification of vascular signals (Figure for Reviewers 4). However, we could not assess the inner and outer vessel wall region quantitatively. It might be possible to quantify whether cancer cells are entering or exiting vessels by skeletonizing them and defining their lumen, as

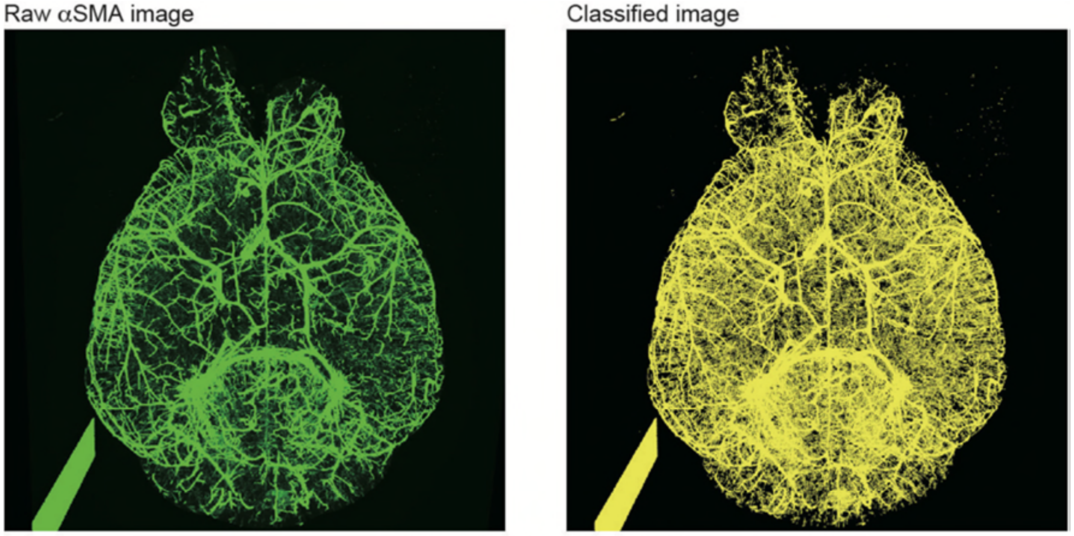

Figure for Reviewers 4

reported by Kirst et al. in 2020.

Figure 2:

3) The aSMA stain is difficult to see as two images in 2A.

According to the referee's comment, we added the caption above the image (up, second from the left) in Fig. 2b in the revised version (Fig. 2a in the original version).

Figure 3:

4) Can the authors quantitate the metastatic volume in the parenchyma vs vessels? Can micromets vs mets with more than 50 cells be distinguished?

We appreciate the referee's comment. As we stated in our response to Specific questions 2) in Figure 1, we are currently unsuccessful in the quantification regarding whether cancer cells are inside or outside of the vessels. With regard to the distinction between micrometastasis and metastasis, we tried to address it by taking nuclear staining images and quantifying how many nuclei are present in each metastatic foci (Figure for Reviewers 5). However, it is difficult to validate the results by the histological examinations. Thus, we would like not to mention it in the manuscript.

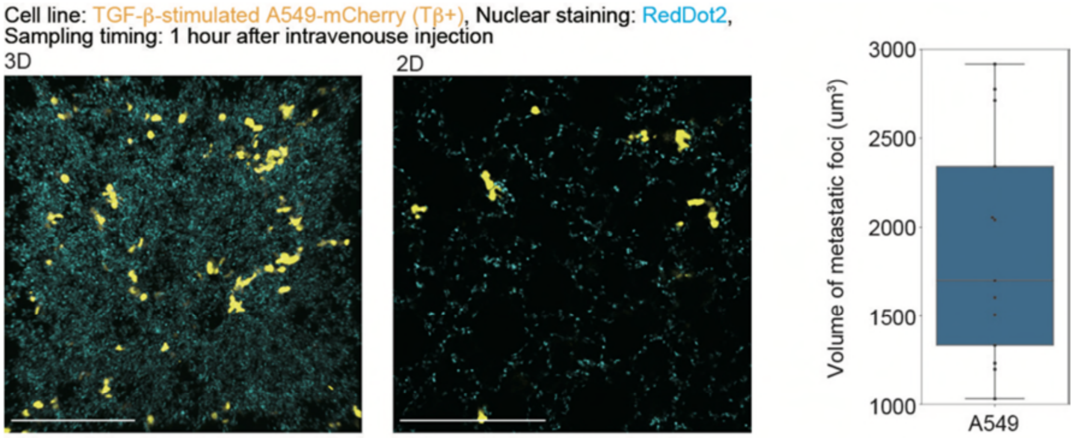

Figure for Reviewers 5

Figure 4:

5) Is there a difference between right or left lungs? Do specific lungs have different regional enhancements? Does the tissue clearing work better for larger or smaller lung tissue?

We appreciate the referee's comment. Distribution of lung metastatic colonies was

examined in the right or left lobe. In our preliminary analysis, there were no significant differences between the left lobes and the right lobes (Figure for Reviewers 6). Because these analyses are preliminary, we would like not to mention it in the manuscript. As regard to lung size in tissue clearing, it is easier in the smaller lungs. However, we took enough time to clear all kinds of organs.

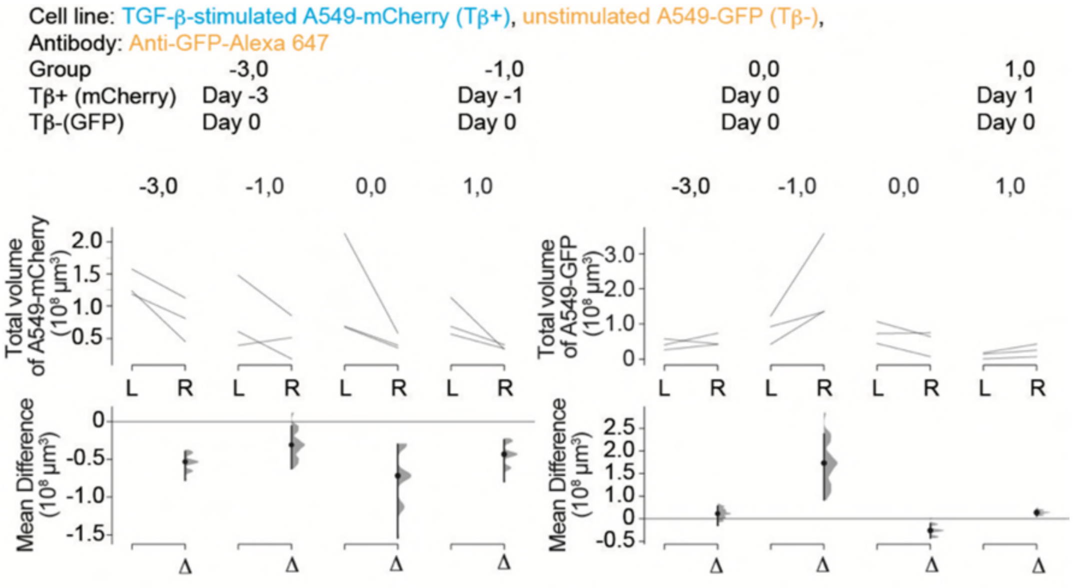

Figure for Reviewers 6

Figure 5:

6) Were there any unexpected TGFbeta responses or signaling pathways? Do specific known mutations in A549 human lung cancer cells cause TGFbeta response to lose growth control or other switching from tumor suppression to promotion?

We appreciate the referee’s fruitful suggestion. Since we have already reported that *PDGFB* is one of the targets of TGF-β, these results were not unexpected. TGF-β is known to induce growth of some normal and cancer cells through induction of PDGF-A or B; however, TGF-β stimulation inhibits growth of A549 cells (Matsuyama et al., ***Cancer Res.***, 2003 and Battegay et al., ***Cell***, 1990). As far as we know, there are no known mutations specific to A549 cells. We thus added some additional descriptions in the revised manuscript (Discussion section (main text, page 17, line 12-15)).

Figure 6:

7) Could the authors expand on the platelet mechanism for a source of activated TGFbeta and how this strategy compares with other TGFbeta targeting approaches in

greater depth?

We appreciate the referee’s fruitful suggestion. We think that platelets also function as a source of TGF-β, and it should be important to consider platelet function when designing the therapies targeting TGF-β. We added these detailed descriptions in the revised manuscript (Discussion section (main text, page 17, line 15-20)).

REVIEWERS' COMMENTS:

Reviewer #1 (Remarks to the Author):

All my concerns were satisfactorily addressed by the authors. I suggest acceptance for publication.

My only suggestion is to provide more detail on the box plots shown in Supplementary Figs. 1a and 2. (what do the boxes represent? standard deviation? a specific percentile? what are the dots?)

Reviewer #2 (Remarks to the Author):

Thank you for your consideration of my suggestions and for improving the manuscript.

Dear Reviewers,

We would like to thank the reviewers for constructive comments to our current work. We have taken the suggestion into account in the revised version of our paper. We listed one-by-one response below.

Reviewer #1

All my concerns were satisfactorily addressed by the authors. I suggest acceptance for publication.

My only suggestion is to provide more detail on the box plots shown in Supplementary Figs. 1a and 2. (what do the boxes represent? standard deviation? a specific percentile? what are the dots?)

We appreciate the reviewer for raising an important point. The box-and-whisker plot represent the minimum, the maximum, the median, and the first and third quartiles of the dataset. The dots in Supplementary Fig 1a represent the value of coefficiency calculations on a single dataset. The dots in Supplementary Fig 2 represent the exact value of each experimental result. We added these data in the revised manuscript (Supplementary Fig. 1 (page 2, line 8-10), Supplementary Fig. 2 (page 4, line 5-7)).

Reviewer #2

Thank you for your consideration of my suggestions and for improving the manuscript.

We are thankful for the time you expended.
