# Supplementary material for: Whole-organ analysis of TGF-β-mediated remodelling of the tumour microenvironment by tissue clearing
Source: Commun Biol. 2021 Mar 5;4:294. doi: 10.1038/s42003-021-01786-y (PMC7935961; doi:10.1038/s42003-021-01786-y)
Supplement: Supplementary file 2 — Peer Review File [file 42003_2021_1786_MOESM2_ESM.pdf]

Supplementary Information

Whole-organ analysis of TGF-β-mediated remodelling of the tumour microenvironment by tissue clearing

Shimpei I. Kubota[1], Kei Takahashi[1], Tomoyuki Mano[2], Katsuhiko Matsumoto[3], Takahiro Katsumata[2], Shoi Shi[2], Kazuki Tainaka[4], Hiroki R. Ueda[2, 3], Shogo Ehata[1, 5], and Kohei Miyazono[1]

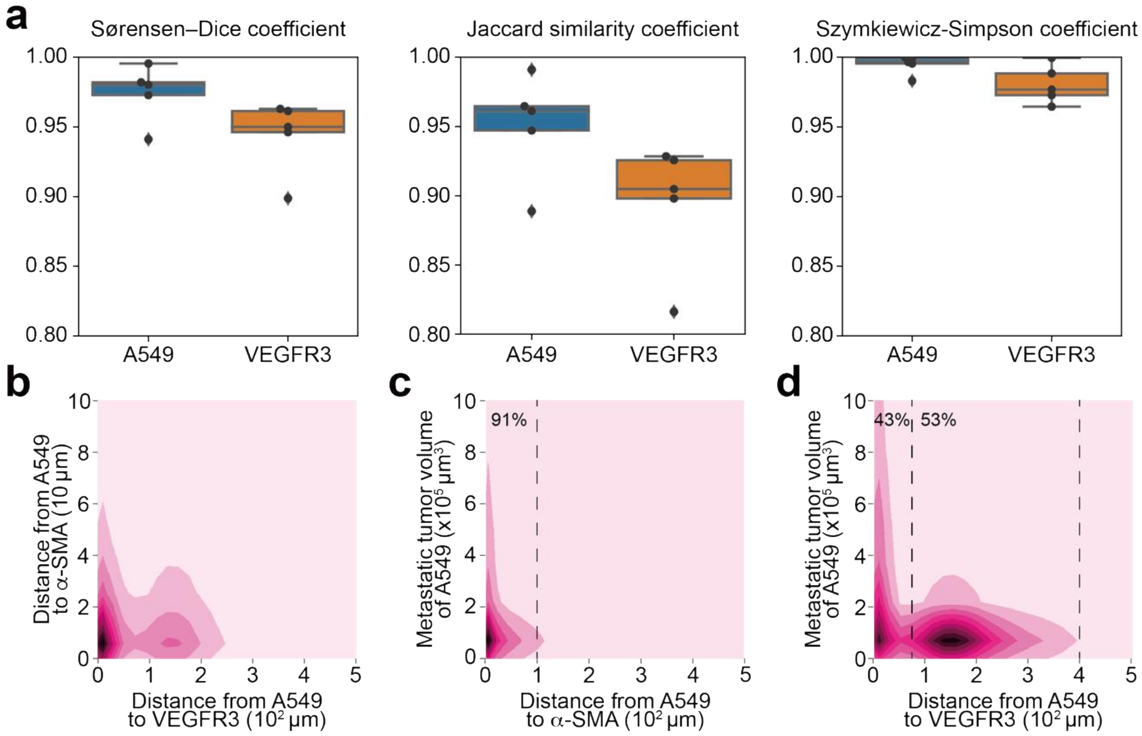

Supplementary Figure 1. Analysis of intercellular distance using whole-organ clearing protocol.

(**a**) Evaluation of our analytical pipeline with several coefficient scxores (Sørensen-Dice coefficient, Jaccard similarity coefficient, and Szymkiewicz-Simpson coefficient) between two binary sets: the ground truth by manual annotation and the prediction by this paper's analysis pipeline for A549 and VEGFR3 signals. The box-and-whisker plot represent the minimum, the maximum, the median, and the first and third quartiles of the dataset. (**b-d**) Spatial analysis of cancer cells and the tumour microenvironment components in lung of mice in Fig. 2. The distribution plot of the distance between A549 cells and α-SMA-positive vessels and between cancer cells and VEGFR3-positve vessels are shown in (**b**). The density plot of the volume of metastasis and the distance

from α-SMA-positive vessels are shown in (**c**). The density plot of the volume of metastasis and the distance from VEGFR3-positive vessels are shown in (**d**).

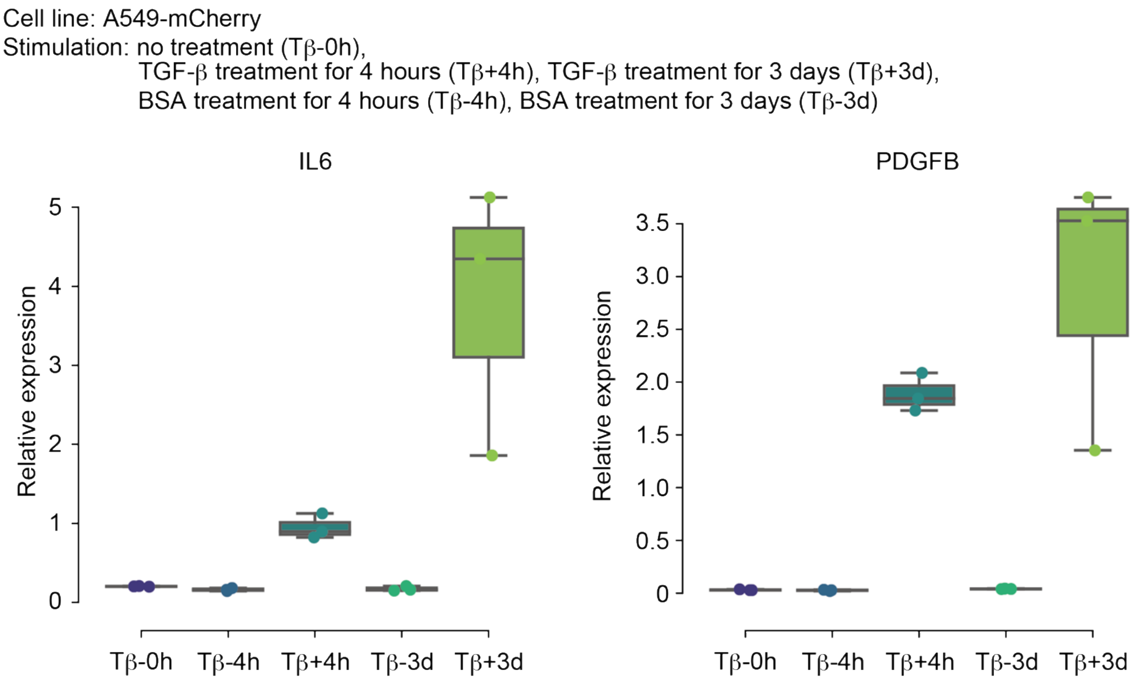

Supplementary Figure 2. Analysis of the up-regulated genes by TGF-β.

A549 cells were stimulated with or without TGF-β1 (5 ng ml^{-1}) for 4 hours or 3 days. Relative expression levels of IL6 and PDGFB mRNA are shown. The box-and-whisker plot represent the minimum, the maximum, the median, and the first and third quartiles of the dataset.

Supplementary Table 1. List of antibodies used in immunostaining.

Indicated antibodies were used for immunostaining in the present study.

**First reaction antibody**	**Clone number**	**Catalog number**	**Company**	**Dilution**	**Sampling day after cancer cell injections**
FITC-conjugated anti-α-SMA antibody	1A4	F3777	Sigma-Aldrich Co. LLC, St. Louis, MO	1:200	1 hour (Fig. 6d), 7 days (Fig. 1c, Fig. 2)
FITC-conjugated anti-Ki-67 antibody	SolA15	11-5698-82	Invitrogen, Waltham, MA	1:100	14 days (Fig. 1c)
Alexa Fluor 647-conjugated anti-GFP antibody	FM264G	3380006	Biolegend, San Diego, CA	1:100	1 hour (Fig. 6a), 14 days (Fig. 3, Fig. 4)
Red Fluorochrome(635) -conjugated anti-Iba1 antibody	-	013-26471	FUJIFILM Wako Pure Chemical Corporation, Osaka, Japan	1:100	1 day (Fig. 7b, Fig. 7e), 14 days (Fig. 1c)
Anti-VEGFR3 antibody	-	AF743	R&D SYSTEMS, Minneapolis, MN	1:100	1 day (Fig. 1c, Fig. 7e), 7 days (Fig. 2)

**Second reaction antibody**	**Clone number**	**Catalog number**	**Company**	**Dilution**	**Sampling day after cancer cell injections**
Alexa Fluor 546-conjugated anti-Goat IgG (H+L) cross-adsorbed secondary antibody	-	A-11056	Thermo Fisher Scientific, Waltham, MA	1:200	1 day (Fig. 1c, Fig. 7e), 7 days (Fig. 2)

Supplementary Table 2. Primer sequences used in the present study.

Indicated primers were used for qRT-PCR analysis in the present study.

Gene	Orientation	Sequence (5' to 3')
IL6	Forward	CCTGAACCTTCCAAAGATGGC
	Reverse	TTCACCAGGCAAGTCTCCTCA
PDGFB	Forward	CTGTCTCTCTGCTGCTACCTG
	Reverse	TCGAGTGGTCACTCAGCATC
HPRT1	Forward	TTTGCTTTCCTTGGTCAGGC
	Reverse	GCTTGCGACCTTGACCATCT
